# The aryl hydrocarbon receptor is associated with monocytic AML and innate immune resistance reversible with an AHR inhibitor

**DOI:** 10.3389/fimmu.2025.1554166

**Published:** 2025-12-09

**Authors:** Jennifer N. Saultz, Daniel Bottomly, Faith Burns, Kaelan Byrd, Yoko Kosaka, Bernhard Alber, Daniel Chandra, Stephen E. Kurtz, Guang Fan, Andy Kaempf, Nicola Long, Marta Sanchez-Martin, Lei Wang, Karen McGovern, Dan S. Kaufman, Shannon K. McWeeney, Brian J. Druker, Jeffrey W. Tyner, Evan F. Lind

**Affiliations:** 1 Division of Hematology/Medical Oncology, Oregon Health & Science University, Portland, OR, United States; 2 Knight Cancer Institute, Oregon Health & Science University, Portland OR, United States; 3 Department of Molecular Microbiology and Immunology, Oregon Health and Science University, Portland, OR, United States; 4 Molecular Pathology and Molecular Diagnostics Laboratories, Knight Diagnostic Laboratories, Oregon Health & Science University, Portland, OR, United States; 5 Biostatistics Shared Resource (BSR) Knight Cancer Institute, Oregon Health & Science University, Portland, OR, United States; 6 IKENA Oncology, Boston, MA, United States; 7 Division of Regenerative Medicine, Department of Medicine, University of California, San Diego, La Jolla, CA, United States; 8 Division of Bioinformatics and Computational Biology, Department of Medical Informatics and Clinical Epidemiology, Oregon Health & Science University, Portland, OR, United States; 9 Division of Oncological Sciences Oregon Health & Science University, Portland, OR, United States; 10 Department of Cell, Developmental & Cancer Biology, Oregon Health & Science University, Portland, OR, United States

**Keywords:** leukemia, immunotherapy, NK cells, AhR, AML

## Abstract

**Background:**

Acute myeloid leukemia (AML) is characterized by a complex interplay between genomic alterations, aberrant hematopoiesis, and immune evasion. The aryl hydrocarbon receptor (AHR) pathway is a critical player in this phenomenon determining the fate of stem cell differentiation as well as dictating immune cell development and function. Despite this critical connection, little is known about how AHR regulates the immune microenvironment in AML.

**Methods:**

We performed a retrospective study examining the pre-treatment effect of immune cell numbers (T and NK cells) in the bone marrow and their impact on overall survival in AML patients undergoing 7+3 induction chemotherapy. Utilizing flow cytometry and both bulk and single-cell RNA sequencing of AML patient samples, we characterized the immune signature of blast cells and the influence of AHR on the immune microenvironment. Lastly, we performed functional studies to determine impact of pharmacological and genomic AHR inhibition on NK cell function.

**Results:**

Higher bone marrow NK cell percentage in ND-AML correlated with poorer OS and expression of HLA-E on leukemic blasts. AHR upregulation was associated with HLA-E expression on blasts and an innate immune resistant signature defined by upregulation of key cytokine pathways, interferon gamma (IFN-g) pathway, and MHC class I/II as well as impaired NK cell profiles. High AHR expression in AML was associated with monocytic maturation and discrepant MHC class I/II profiles. Pre-treatment of blasts with an AHR inhibitor (AHRi) prior to NK cell killing assay downregulated key checkpoint molecules, including HLA-E, and key IFN-g signaling transcription factors (STAT1, IRF1) and led to enhanced NK cell killing among multiple FAB subsets in AML.

**Conclusion:**

The data support targeting the AHR pathway as a dual tumor intrinsic and immune targeting therapeutic strategy for AML, particularly in combination with NK cellular therapy.

## Introduction

Acute myeloid leukemia (AML) is a highly lethal disease characterized by clonal, neoplastic myeloid expansion and resultant bone marrow dysfunction. Despite significant advancements in our knowledge and treatment of AML, tumor-driven resistance strategies and crosstalk between immune cells remains elusive. As leukemic blasts progress, innate and adaptive immune resistance signatures emerge, highlighting the dual role of mutation acquisition and immune tolerance (1). Natural killer (NK) cells are a potential “off the shelf” cell-based therapy capable of inducing long-term survival in hematological malignancies (2–4). NK cell function relies on the complex interplay between activating and inhibitory signals. Key NK cell activating receptor-ligand pairs include NKG2D receptors (MICA, MICB, ULBP1, ULBP2/5/6), and FAS, while the presence of HLA class I, LILRB4, B7H3, TRAIL1, PD-L1, HLA-E and PD-L2 are known to inhibit NK cell function (5–7). Immature NK cells (i.e., Stage 1–3 including early progenitors which are CD56 negative, CD94 negative and CD16 negative produce higher levels of cytokines with little to no cytotoxicity while more mature NK cells (i.e., Stage CD56 bright/CD94 positive, low to absent killer immunoglobulin-like receptor (KIR), high inhibitory CD94/NKG2A heterodimer positive and Stage 5 CD56dim, CD94 negative, high KIR, and CD16 positive) have anti-tumor natural cytotoxicity as well as antibody dependent cellular cytotoxicity (ADCC) in the presence of CD16 expression (8). Major histocompatibility complex class I (MHC-I) HLA ligand expression on healthy and leukemic blasts mediate immune recognition. Downregulation of MHC-I classical (HLA-A, -B and -C) in AML leads to impaired CD8^+^ T -cell recognition, however, may increase susceptibility to NK cell mediated killing through lack of engagement with KIR and CD94/NKG2A heterodimer, respectively (9–13). MHC class II and non-classical MHC class I expression (HLA-E/F/G) also play major roles in immune recognition (14, 15). Previous studies have shown the NKG2A-HLA-E axis as a critical induced checkpoint for cytokine-induced memory-like NK cells leading to treatment failure in patients with AML (16). HLA-E, as well as other MHC class I genes, have been previously shown to be induced by IFN-γ, although HLA-E appears to be most sensitive based on the presence of a distinct IFN-γ-responsive element, termed the interferon response region (IRR) (17). In AML, u of HLA-E on leukemic blasts has been shown to occur through secretion of immature NK cell and T cell production of IFN-γ leading to leukemic blast immune evasion (18).

The aryl hydrocarbon receptor (AHR) is a ligand-activated transcription factor that regulates immune responses, cell growth, differentiation and environmental chemical detoxification pathways. AHR has many activating ligands, including 2,3,7,8-Tetrachlorodibenzo-p-dioxin (TCCD), formylindolo [3,2-b] carbazole (FICZ), metabolites of arachidonic acid, dietary indoles (cruciferous vegetables), kynurenine pathway and catabolism of tryptophan to N-formyl-kynurenine through tryptophan-2,3-dioxygenase (TDO2) and indoleamine-2,3-dioxygenases (IDO1 and 2) (19). Once AHR is activated, it dimerizes with AHR nuclear translocator (ARNT) to induce *AHR*-regulated genes including Cytochrome P450 1A1 (*CYP1A1)*, Cytochrome P450 1B1 (*CYP1B1)* and Thrombospondin-1 (*THBS1)* by binding directly to gene promoters. Downstream effects of AHR activation include upregulation of MAPK and STAT signaling, cell differentiation, and regulation of immune cell maturation (20). Specifically, the AHR/kynurenine pathway is closely regulated by IFN-γ, which plays a central role in immune responses and inflammation. IFN-γ strongly induces the expression of IDO1, which catalyzes the conversion of tryptophan into kynurenine (21). This shift in tryptophan metabolism leads to local tryptophan depletion and kynurenine accumulation. Kynurenine acts as a ligand for AHR, promoting the differentiation of regulatory T cells (Tregs) and suppressing effector T cell activity, contributing to immune tolerance (22). Elevated kynurenine levels and kynurenine/tryptophan ratios are frequently observed in conditions characterized by chronic IFN-γ signaling, such as infections, cancer, and autoimmune disorders (23). Therefore, the kynurenine pathway serves as a key downstream effector of IFN-γ signaling, translating inflammatory cues into metabolic and immune regulation that can either resolve inflammation or facilitate immune evasion, depending on the context.

In AML, AHR has been found to be a driver of monocytic differentiation and chemotherapy resistance (24) (25, 26). In human and murine AML, AHR activation impairs innate lymphoid cell (ILC) development, blocking maturation, while AHR inhibition restored a normal NK cell maturation profile and sensitized leukemic blasts to NK cell-mediated killing (27, 28). The mechanism of enhanced killing with AHR antagonist has not been fully elucidated. Herein, we report a connection between AHR activation and high IFN-γ signature, monocytic differentiation, HLA-E expression and a resultant innate immune resistance. This unique axis is associated with high IFN-γ receptor 1 (*IFNGR1*) gene signaling and differential expression of key NK cell receptor-ligand pairs. Inhibition of AHR prior to NK cell co-culture resulted in enhanced NK cell killing among many FAB AML subsets and downregulation of STAT1. Our data adds to the existing literature that inhibiting AHR may be a therapeutic strategy to augment leukemic blast sensitivity to NK cellular therapy through modulation of HLA-E.

## Materials and methods

### Cell culture

Primary peripheral blood and marrow mononuclear cells (PBMCs) were obtained from healthy donor and AML patients in accordance with the Oregon Health & Science University Institutional Review Board (OHSU IRB protocol 4422) and the Declaration of Helsinki. The human MOLM-14 cell line (FLT3-ITD positive) were obtained from ATCC. STR profiling for human cell line authentication was performed in the OHSU DNA Services Core and tested for mycoplasma on a monthly schedule. All cell lines were maintained in RPMI, 10-20% fetal bovine serum (FBS), L-glutamine and penicillin/streptomycin. IK-364 was obtained from IKENA Oncology. Chemical structure is shown (**Supplementary Figure 1A**). The starting dose of IK-364 (3 µM) was chosen after testing percent inhibition of viable cells across graded concentrations on MOLM-14 parental cell line (**Supplementary Figure 1B**), and chosen based on inhibition of CYP1B1, a gene which is regulated by AHR (**Supplementary Figure 1C**) Primary AML PBMCs, previously frozen, were thawed and assayed in RPMI media containing 20% fetal bovine serum, 1% antibiotic/antimycotic, human interleukin-3 (hIL-3; 10ng/mL), hIL-2 (100 U/mL), hIL-6 (100 ng/mL) and stem cell factor (100 ng/mL). Primary patient samples were plated at 2 x 10^6^cells/mL and cell lines were co-cultured at a concentration of 0.5 x 10^6^ cells/mL in 2mL of media with IK-364 at 500 nM, 3 µM or DMSO (vehicle control) for 1 day, 2 days or 3 days, prior to performing assays. Annexin stain was used to determine initial viability between DMSO and treated samples without direct cell death noted at either 500 nM or 3 µM IK-364 dosing for 24 hours in healthy donor or AML patient samples (**Supplementary Figure 1E**).

### Flow cytometry

Briefly, 1 x 10^6^ cells were stained with zombie aqua (Biolegend, #423101) and incubated for 15 minutes at room temperature in the dark. Cells were then washed with FACS buffer (PBS 2% bovine calf serum, 0.005% sodium azide) and resuspended in 20 μL/sample 1:50 human Fc block (Biolegend #422302) and incubated for 5 minutes on ice. Antibody cocktail was added to each sample for 30 minutes, washed and resuspended in FACS buffer prior to running on a BD Fortessa or BD FACSAria. Data analysis was done using FlowJo software. Antibodies used to characterize NK cell profiles are found in **Supplementary Table 2**.

### RT-PCR for CYP1B1

RNA was isolated utilizing the PureLink RNA Kit (Life Technologies). Reverse transcription of RNA was performed utilizing the Superscript VILO system (LifeTechnologies). Quantitative PCR (qPCR) was performed using primers with SYBR Green Master Mix (Life Technologies) using a ViiA 7 RT-PCR system (Life Technologies) with CYP1B1 and glyceraldehyde-3-phosphate dehydrogenase (GAPDH) (internal control). SYBR Green primers were obtained from IDT. Primers are listed in **Supplementary Table 3**. Gene expression was normalized to an internal control (DCt = Ct gene of interest – Ct internal control).

### Bulk RNAseq analyses

Among 69 newly diagnosed (ND) AML patients treated with 7 + 3 chemotherapy at OHSU (this clinical dataset is further explained below), 39 had available bulk RNA sequencing (RNAseq) data from Beat AML (with the generation and processing of these RNAseq data described in the original Beat AML paper) (29). Of these 39 AML patients, n=10 “low” NK cell (2.4% - 4.4%) and n=10 “high” NK cell (11.1% - 22.6%) were selected based on the bottom and top quartiles, respectively, of NK cell percentage of lymphocytes. Gene-level read counts from Beat AML were exposed to low-count filtering and normalized by the trimmed mean of M-values method (‘edgeR’ Bioconductor/R package). Surrogate variable analysis (‘SVA’ Bioconductor/R package) was applied to address confounding from unmeasured sources. For the 20 included patients, differential expression analysis (DEA) comparing NK high to NK low percentage was performed with the limma-voom procedure (‘limma’ Bioconductor/R package) upon adjusting for specimen type and 2 identified surrogate variables (30). Differentially expressed genes (defined as those with unadjusted p-value < 0.05) were utilized to conduct over-representation analyses within Gene Ontology (GO) terms and KEGG (Kyoto Encyclopedia of Genes and Genomes) pathways (‘limma’ package). Additionally, ordered (by log_2_fold change) gene set enrichment analysis (GSEA) was performed on all Reactome pathways comprising 5 to 500 Entrez ID-annotated genes (‘ReactomePA’ Bioconductor/R package).

Bulk RNAseq-based normalized gene expression values were obtained from Beat AML, with generation of these values previously explained (28, 50). “High” and “low” categorizations for *AHR* and *HLA-E* expression were defined as the top and bottom 10% of corresponding values, respectively. DEA was performed using limma-trend v3.54.1 (31). The resulting T-statistics were used to perform pre-ranked GSEA using ReactomePA v1.42.0 (32). Pathway significance was determined as FDR-adjusted p-value ≤ 0.05 (33).

Additional bulk RNAseq was performed on the MOLM-14 parental cell line, including 3 replicates a piece for 24-hour treatment with DMSO (vehicle control) or IK-364. OHSU’s Massively Parallel Sequencing Shared Resource (MPSSR) performed library preparation (TruSeq Stranded mRNA kit; Illumina Inc.), sequencing (NovaSeq 6000; Illumina), base calling (RTA v3.4.4; Illumina), paired-end read trimming (Trimmomatic 0.36), and alignment to the GRCh38.89 reference genome. Gene-level read counts were filtered (removing lowly-expressed genes) and normalized by the Trimmed Mean of M-values method (‘edgeR’ R/Bioconductor package). Surrogate variable analysis identified unmeasured confounders to be controlled for as covariates in gene-wise models. The limma-voom procedure (‘limma’ R/Bioconductor package) was applied to each remaining gene (using empirical Bayes to borrow information between genes) for DEA of IK-364 vs. DMSO, which produced log_2_ fold change estimates and associated raw and FDR-adjusted p-values (34).

### CRISPR-Cas9 single gene inactivation by individual sgRNAs

Single sgRNA sequences were designed using Synthego design tool (https://design.synthego.com/#/) and converted to DNA sequences. Phosphorylated complementary oligonucleotides were annealed and ligated into BsmbI-digested pLentiCRISPRV2 backbone, containing sequences for Cas9 and puromycin resistance, and then validated by Sanger sequencing. Lipofectamine 2000 (Invitrogen, #11668019) was used to transfect HEK293T cells with single transfer vectors with packaging plasmids psPax2 (Addgene, #12260, RRID: Addgene_12260) and VSVG (Invitrogen) to generate virus. Viral supernatants were collected, filtered through 0.45-µm filters, and used for transduction of MOLM-14 cells using a spinoculation method. Knockdown of *AHR* and *IFNGR1* was carried out by cloning sgRNAs into plentiCRISPRv2 (Addgene, #52961) as described previously (35). All sgRNAs used in this study are provided in **Supplementary Table 5**. Cells were selected with 2µg/mL of puromycin for 5 to 7 days and grown for 14 days in culture before experiments.

### Western blotting

For the IFN-γ experiments, 2 x 10^6^ MOLM-14 cells were seeded in 4ml of RPMI with 100 IU/ml IFN-γ or 0.1% BSA in PBS. Following overnight incubation, cells were pelleted and lysed. Protein concentration was calculated with a BCA protein assay kit (Thermo Fisher). 120µg protein were mixed with NuPAGE LDS Sample Buffer (Invitrogen) and NuPAGE Sample reducing Agents and denatured for 10min at 95°C. Cell lysates were loaded onto each lane of a 4-15% Criterion Tris-HCl Protein gel (BioRad) and transferred onto a PVDF membrane (Millipore). Membranes were incubated in primary antibody at 4°C overnight prior to incubation in secondary antibody. ECL reagent (BioRad) was used to visualize protein. The primary antibodies included anti-AHR (Cell Signaling Technologies #83200). The protein levels were normalized to β-tubulin (Cell Signaling Technologies #2146).

### Single-cell RNA sequencing

Five previously frozen peripheral blood AML mononuclear cells were selected for single cell sequencing based on normalized log2 RNA expression and available samples (2 AHR high >8 and 3 AHR low <5). Samples were thawed and sorted for viable cells at the OHSU Flow Cytometry Core Facility. Single-cell RNA sequencing was performed by the OHSU Massively Parallel Sequencing Shared Resource using the 10x Genomics Chromium system with Single Cell 3’ v3 chemistry. Alignment and counting were performed using CellRanger v6.1.2 relative to GRCh38. We removed cells with overall counts < 1,000 or > 20% mitochondrial gene count percentage. Additionally, we removed all cells flagged as doublets by the `scDblFinder` package (36) separately for each sample. We initially annotated the cells using an adapted version of the RandomForest classifier described previously (37). Processing was performed using Seurat with integration using Harmony. Pseudo-bulk differential expression was performed using limma-voom after removing samples/conditions with fewer than 10 cells (31). FDR adjustment was performed using qvalue (38).

### Analysis of single-cell NK and T cell subsets

We first selected NK cells by requiring CD3D, CD3E and CD3G to be zero and either NKp80/KLRF1 or CD56/NCAM1 to be greater than zero as previously proposed (39). We then clustered these cells using Leiden clustering in Seurat after integrating the samples using Harmony. We used a small resolution (0.25) to coarsely define clusters. From examining these clusters, we identified one containing high quality NK cells based on expression of known markers (CD7, KLRF1, NKG7, GNLY) (40). We further stratified these cells into four groups based on the non-zero expression of CD16/FCGR3A and CD94/KLRD1. We evaluated these four groups for differential abundance using edgeR (41, 42). Similarly for T-cells, we first selected cells that had expression of at least one of the CD3 subunits. We then clustered these cells using Leiden clustering in Seurat after integrating the samples using Harmony. Selecting for fewer but larger clusters we used a Leiden resolution of 0.25. We selected two clusters based on the expression of the CD4 and CD8A and B genes, respectively.

### Statistical analyses

Group differences were tested for continuous features with the Wilcoxon rank sum test and for categorical features with Fisher’s exact test. Specific statistical analyses are mentioned in other (context relevant) subsections of the Methods and described in figure legends. In all figures, “ns” denotes not significant (P ≥ 0.05), “*” denotes *P* < 0.05, “**” denotes *P* < 0.01, “***” denotes *P* < 0.001, and “****” denotes *P* < 0.0001.

### Clinical outcome association with bone marrow NK cell percentage

Analysis was performed on a clinical cohort comprised of 69 adults with newly diagnosed AML (including 2 patients with an antecedent hematological disorder and 1 with therapy-related AML) between September 2010 and March 2016 who had a diagnostic bone marrow biopsy that underwent clinical flow cytometry and targeted next-generation sequencing (NGS) within 30 days prior to 7 + 3 induction treatment at OHSU on an IRB-approved protocol. Multicolor flow cytometry performed on the patient’s bone marrow yielded cellular estimates including white blood cell (WBC) count, leukemic blast percentage, lymphocyte percentage, absolute NK cell count (cells per microliter), and NK cell percentage (of lymphocytes). Lymphocytes were identified by CD45 expression and light side scatter and NK cells were defined as CD20^-^, CD14^-^, CD3^-^, and CD56^+^. NK cell percentage was dichotomized at the cohort median, resulting in “low” (n = 35; range = 2.1% – 8.7%) and “high” (n = 34, range = 8.8% – 22.6%) NK groups. Overall survival (OS) was measured from AML diagnosis date to the date of death or last contact, estimated with the Kaplan–Meier method, and compared between NK groups using the log-rank test and Cox regression models that produced hazard ratios and associated p-values. The effect of bone marrow T cell percentage on OS in a cohort that included these 69 AML patients was previously published.

### NK cell cytotoxicity

For cytotoxicity assays, target cells (MOLM-14 cells or primary patient leukemic blasts) were pre-stained with 1µL of CellTrace™ Violet (Thermo Fisher) at 1:2000 dilution in PBS. Cells were stained at a concentration of 1 x 10^6^ cells/mL for 15 min at 37°C, protected from light. After 15 minutes, 5x cell staining volumes of RPMI-10% FBS was added and incubated for 5 minutes at 37°C. Cells were then pelleted and resuspended in fresh pre-warmed culture medium at 20,000 cells per 50 µL. Previously frozen healthy donor NK cells were thawed and stimulated overnight with 2.5 ng/mL hIL-2 and 1% NEAA (non-essential amino acids) prior to co-cultures. Targets were co-cultured with NK cells at effector-to-target (E:T) ratios of 20:1, 10:1, 5:1, 2.5:1 and 1:1 and incubated at 37°C for 3.5 hours. Caspase-3/7 Green Detection Reagent (Thermo Fisher) and SYTOX AADvanced dead cell stain solution (Thermo Fisher) were added during the last 30 min of culture for a total incubation time of 4 hours. Cells were then analyzed by flow cytometry. NK cell killing was calculated by subtracting the background of untreated target cells from all the other samples of the same experimental group.

### Isolation and expansion of NK cells

NK cells from healthy donor peripheral blood were enriched using the RosetteSep™ Human NK Cell Enrichment Cocktail (StemCell Technologies) which removes unwanted cells via tetrameric antibody complexes recognizing non-NK cells and red blood cells (RBCs). These cells were then expanded on irradiated (100 cGy) K562-IL21 feeder cells (CSTX002, kindly shared by CYTOSEN) (43) at a ratio of 1:2 (PBMC∶ CSTX002) in NK cell media at 2×10^5^ PBMC/mL. Cultures were refreshed with half-volume media changes every two to three days and re-stimulated with CSTX002at a ratio of 1∶1 every seven days. Expanded NK cells were carried forward for subsequent stimulations or cryopreserved for later experiments.

## Results

### AML overall survival is associated with bone marrow NK cell percentage at the time of diagnosis

**Figure 1:**
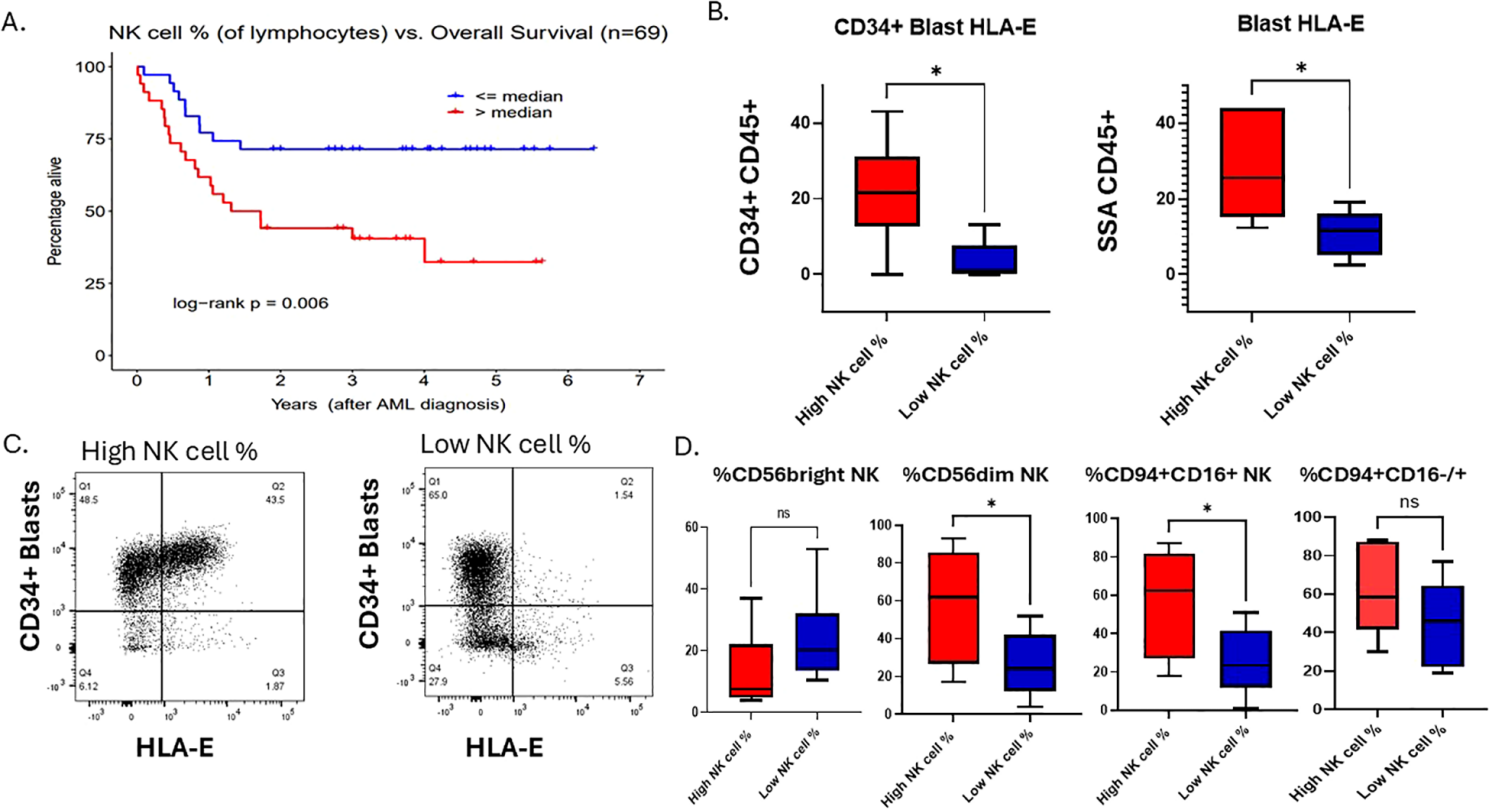
AML overall survival is associated with bone marrow NK cell percentage at the time of diagnosis. **(A)** Overall survival Kaplan-Meier curves for “low” vs. “high” total NK cell % groups as determined by the sample median. **(B)** Flow cytometry analysis of AML blasts gated on CD34+ HLA-E+ or SSA/CD45+ blasts, respectively, of NK cell % high patient and NK cell % low patient dichotomized at the cohort median. Student t test, *P <.05. Error bars indicate SD. **(C)** A flow cytometry plot showing HLA-E on x-axis and CD34+ on the y-axis of a representative patent from the high NK cell % cohort and one from the low NK cell % cohort. **(D)** Flow cytometry analysis of 12 primary AML samples within our clinical cohort with relative % of NK cell subsets of lymphocytes that are CD56 bright, CD56dim NK cells or CD94+/CD16+/-, gated on lineage negative (CD3, CD20, CD14) in NK% high (n=6) and NK% low (n=6).

We conducted a retrospective study n=69 newly diagnosed (ND) adult AML patients who underwent intensive chemotherapy to determine the influence of pre-treatment NK cell percentage on survival. Patient demographics, disease characteristics, and clinical response- stratified by our median-dichotomized NK cell % groups- are displayed in **Supplementary Table 1**. We found that a higher NK cell percentage was associated with significantly shorter OS (Hazard Ratio [HR]=1.10 for each 1-unit increase in NK%, p=0.002; HR = 2.78 for NK% >median vs. ≤median, p=0.006; **Figure 1A**) similar to other publications (44, 45). This negative impact of higher NK% was also observed when adjusting for age, ELN risk, and *de novo* vs. secondary AML status (adjusted HR = 1.08, p=0.026 for each 1-unit increase; adjusted HR = 2.06, p=0.086 for NK% >median). While neither the number of marrow T cells (per µL) nor its percentage among lymphocytes was associated with NK% group, a higher T cell percentage prior to intensive induction chemo was correlated with longer survival (1-unit increase in T cell % HR=0.96, p=0.004) (**Supplementary Table 1**). A non-significant trend (p=0.197) toward higher NK% in non-monocytic AML (FAB M0-M2, n=41) with median (interquartile range [IQR]) of 9.1% (6.1%-13.1%) compared to monocytic AML (FAB M4/M5, n=25) with median (IQR) of 7.8% (4.7%-10.1%) suggests cell differentiation state may influence immune profiles. To determine global immune changes within this cohort on a broader scale, we performed RNA sequencing analysis of Beat AML patients who overlapped with our clinical cohort to explore MHC class I and II changes. There were 39 out of 69 patients in our clinical cohort with RNA sequencing. Within this subset of our clinical cohort, we performed DEA to compare n=10 low NK% to n=10 high NK % patients. We observed upregulation of genes encoding MHC class II proteins in our low NK cell % group, specifically: HLA-DRB1 (high to low NK % log2 FC = -2.0, p<0.001), HLA-DPB1 (log2 FC = -1.4, p<0.001), HLA-DPB2 (log2 FC = -3.5, p<0.001), HLA-DPA1 (log2 FC = -1.3, p=0.001), and HLA-DRA (log2 FC = -1.2, p=0.001) (**Supplementary Figure 1D**). Reactome-based GSEA also showed upregulation of the ‘MHC class II antigen presentation’ pathway (comprising ~100 genes) for low NK% patients compared to high NK% (FDR p=0.015). Together, our data suggests that tumor MHC expression may impact immune microenvironment which may reflect leukemic blast differentiation state.

To examine this further, we identified 12 samples with viably frozen bone marrow cells (6 with high NK cell percentage; 6 with low NK cell percentage). We examined cell phenotypes using flow cytometry markers that included CD45, CD34, CD56, CD94, CD16, HLA-E, CD3, CD13, CD14, CD20 and a viability dye. The samples with high NK cell percentage exhibited increased expression of HLA-E on both CD34+ leukemic blasts as well as on the total leukemic blast population (**Figures 1B**, **C**), suggesting that despite a high percentage of mature NK cells, the killing of leukemic blast targets by NK cells may be blocked by inhibitory HLA-E expression on the AML tumor cells. Previously published data shows that overexpression of HLA-E is dependent on IFN-γ produced by immature (CD56 bright) NK cells and CD8 T cells (18). Previous publications have found that higher immature NK cell percentage is associated with worse prognosis (46, 47). Thus, we hypothesized that immature NK cells were responsible for the higher levels of HLA-E on leukemic blasts in these samples. In contrast to this hypothesis, we found that the samples with high NK cell percentage had lower levels of CD56bright NK cells (**Figure 1D**), however this was not statistically significant due to low sample size. Since almost all CD56 bright NK cells also express CD94, we performed additional flow analysis on patient samples within our cohort using CD94 in addition to CD56 and CD16. We found that the NK cell high% samples had relatively higher % of total CD94+ NK cells compared to low NK cell% and found that NK cell% high patients had higher expression of CD94+/CD16+ compared to NK cell% low samples (**Figure 1D**). The CD94+ CD16-/+ NK cells (stage 4/early stage 5) represent a functionally distinct population of early mature, cytotoxic NK cells that play significant roles in interferon gamma production, especially under conditions of activation. Resting CD94+CD16+ NK cells have limited interferon gamma but upon activation by tumor cells produce high levels of interferon gamma. The fact that high NK cell % patients have higher relative numbers of CD94+ CD16+/- NK cells could explain a link between interferon gamma secretion from the microenvironment and higher HLA-E expression on myeloid blasts. Overall, this suggests that NK cell immune profiles in AML patient’s bone marrow at diagnosis impact survival but also may correlate with tumor resistance signatures dependent on leukemic blast maturation state. To first understand the clinical significance of higher HLA-E expression in AML and differentiation state, we set out to determine the genetic signature associated with *HLA-E* gene expression in AML using the Beat AML dataset and The Cancer Genome Atlas (TCGA).

### Characterization of HLA-E in AML

**Figure 2:**
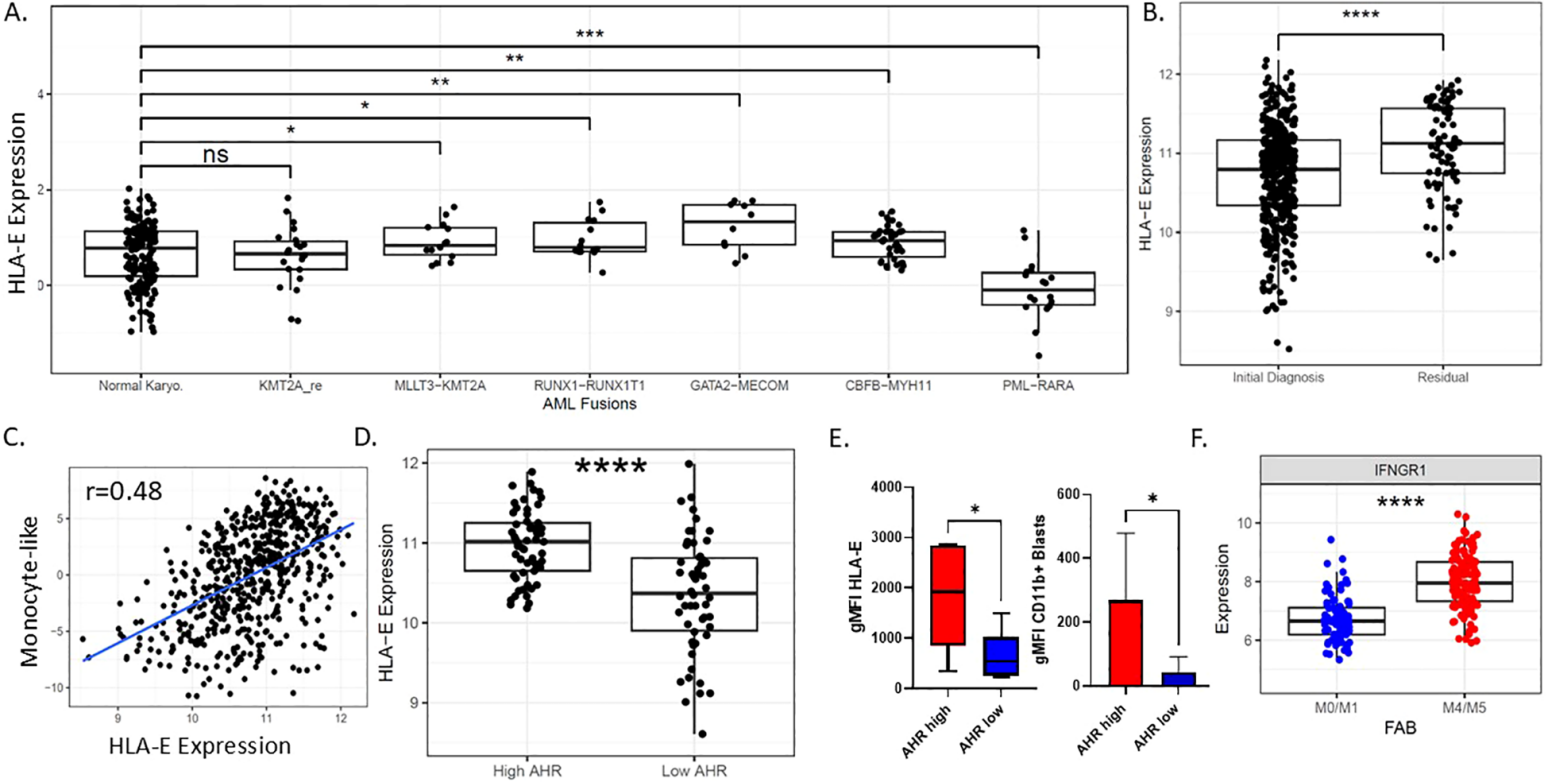
Characterization of HLA-E in AML. **(A)** HLA-E expression by RNA sequencing among common AML genomic fusion partners. **(B)** HLA-E expression by RNA among newly diagnosed and refractory AML patients. **(C)** Pearson correlation plot showing RNA monocyte-like differentiation state scores based on RNAseq as published (32) and HLA-Expression, r=0.48; p-value is 1.075353e-33. **(D)** Expression of RNA levels of HLA-E correlated with AHR RNA expression groups in beat AML patients ****p<0.0001. **(E)** AML patient samples protein expression of HLA-E and CD11b based on AHR low or AHR high status (n=12). **(F)** IFNGR1 correlates with FAB classification state in primary AML samples, ****p<0.0001. * p<0.05, ** p<0.01, *** p<0.001, **** p<0.0001, n.s., Non-significant.

HLA-E is the major ligand for the inhibitory receptor CD94/NKG2A found on both NK cells and subsets of cytotoxic CD8^+^ T cells and is highly conserved among MHC class I genes with only two allelic variants (Arg in HLA-E*0101, Gly in HLA-E*01031) (48). Expression and the role of HLA-E is not well-characterized in AML; however, downregulation can lead to enhanced NK cell recognition. To explore the possibility of targeting HLA-E as a means to enhance cellular therapy, we endeavored to define associations with *HLA-E* RNA expression from Beat AML dataset with ELN risk, genetic subtype and FAB status. *HLA-E* gene expression showed variation across common AML gene fusions; in particular, compared to cytogenetically normal AML, *HLA-E* expression was lowest in promyelocytic leukemia (*PML*)/retinoic acid receptor α (*RARA*) fusion gene (p=0.00016) and highest in *GATA2-MECOM* (p=0.00479) compared to normal karyotype based on the Beat AML dataset (**Figure 2A**) and validated in the TCGA dataset (**Supplementary Figure 4A**). *HLA-E* was upregulated in residual AML versus ND AML, suggesting potential differences in microenvironment favoring immune resistance in AML cases more recalcitrant to up-front therapy (p=<0.00001) (**Figure 2B**). This observation may also suggest that AML therapy can drive an inflammatory process that induces *HLA-E* gene upregulation. We observed a correlation between *HLA-E* gene transcript levels with transcriptional signature classifications of AML cell differentiation state (37). Specifically, we found that *HLA-E* expression was correlated with monocytic-like leukemic blast differentiation state based on RNAseq classification of cell states in both Beat AML dataset by published methods (37) (**Figure 2C**, r=0.48, p-value = 1.075353e-33) and in TCGA dataset **Supplementary Figure 4B** (TCGA monocyte-like vs HLA-E, r=0.517; 0.4-0.617 95% CI; Pval: 1.31e-13; Pearsons correlation). In line with this finding, *HLA-E* was highly correlated with *AHR* (p<0.0001) in the Beat AML dataset, previously found to be a driver of monocytic maturation in AML (25, 26) (p<0.0001) (**Figure 2D**) as well as in the TCGA dataset (High vs Low: 0.69; 0.272-1.11 95% CI; Pval: 0.00207; Welchs T-test) (**Supplementary Figure 4C**). In addition, the protein expression of HLA-E was also found to correlate with AHR levels in primary AML patient samples (**Figure 2E**) (29, 49). Monocytic AML tends to be more inflammatory and may exploit IFN- γ signaling for survival-γ (50). To explain the presence of higher *HLA-E* expression in *AHR* high monocytic AML patient samples, we hypothesized that these tumors may exhibit higher dependence on IFN-γ signaling as dictated by higher relative expression of interferon gamma receptor 1 (*IFNGR1)*. As expected, we found higher *IFNGR1* gene expression in monocytic FAB M4/M5 compared to FAB M0/M1 subtype (**Figure 2F**). The distribution of IFNGR1 among all FAB subtypes is included in **Supplementary Figure 2B**. Since HLA-E can be upregulated by cell stress and high states of IFN-γ (18), we next sought to examine the immunological signatures associated with AHR activation.

### AHR high AML samples have a gene signature enriched in IFN-γ signaling, inflammatory, and immune response pathways

**Figure 3:**
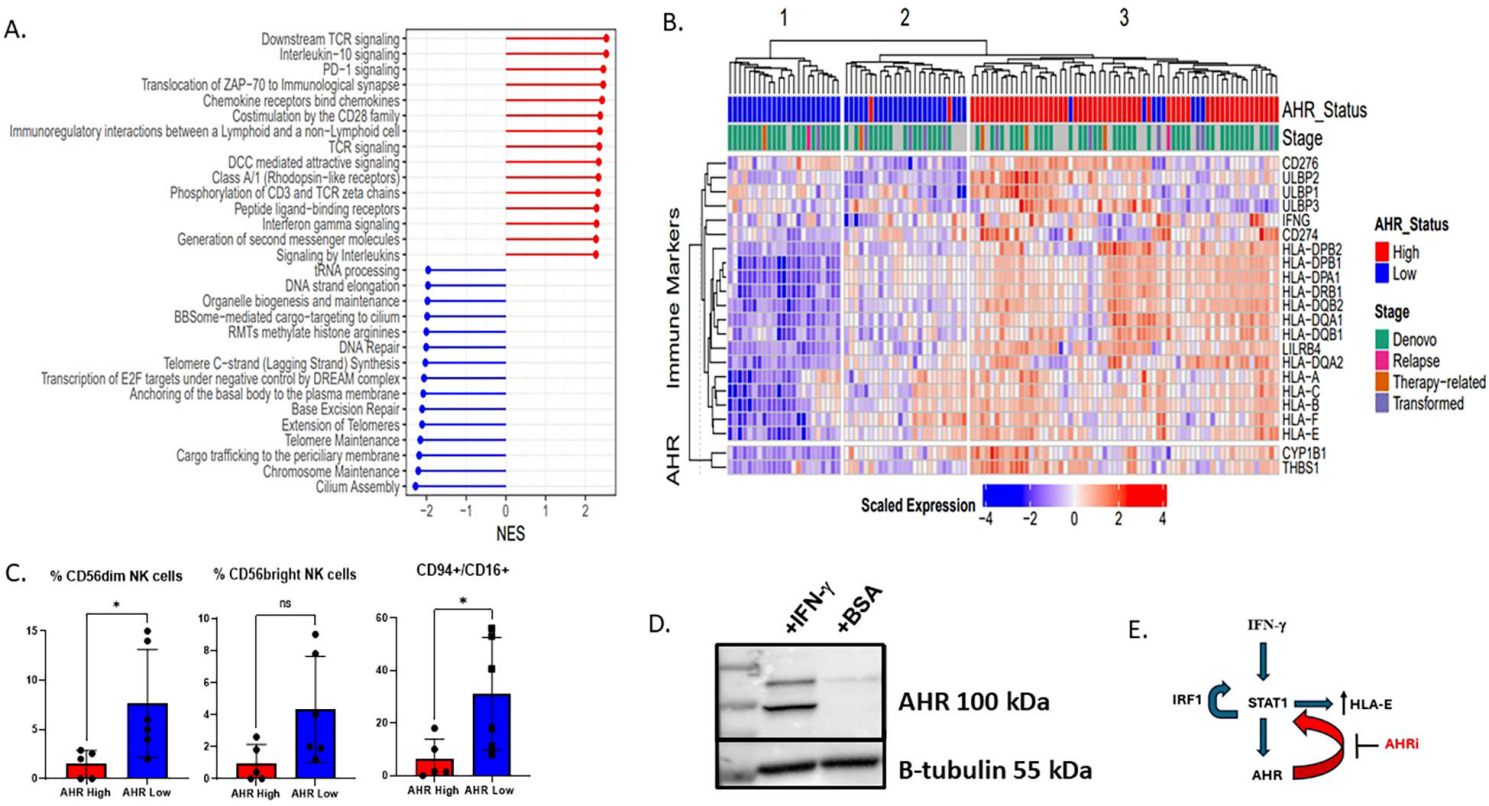
AHR high AML samples have a gene signature enriched for interferon gamma signaling, inflammation, and immune response pathways. **(A)** Forest plot showing the differential expression of key immune pathways in the top 10% AHR high vs bottom 10% AHR low AML samples in beat AML by RNA sequencing. Sample size includes 545 patients. NES is normalized enrichment score between AHR high vs AHR low based on log2 fold change RPKM. **(B)** Heatmap displaying the scaled expression of genes associated with AHR as well as immune ligands. Beat AML patient samples were ranked by AHR with the top 10% deemed high and lowest 10% low. They are shown and clustered based on average-linkage hierarchical clustering (n=111). **(C)** Flow cytometry summary statistics showing 11 patient samples which are AHR high or AHR low (n=11) and relative % of NK cells of lymphocytes based on AHR high (n=5) or low (n=6) on lineage negative CD56+ cells (n=11). * p<0.05 **(D)** MOLM-14 cells treated for 24 hours with interferon gamma 100 IU/ml or BSA (control) and AHR expression was measured by western blot. **(E)** Proposed mechanism of how AHR interacts with HLA-E via STAT1. IFN-γ triggers a signaling cascade involving the IFN-γ receptor and ultimately leads to the phosphorylation of STAT1. STAT1 then can bind to the HLA-E gene promoter, increasing HLA-E transcription and expression. Activation of the JAK/STAT pathway leads to the transcription of IRF1, and IRF1, in turn, can contribute to a positive feedback loop by promoting STAT1 phosphorylation and DNA binding, further enhancing the JAK/STAT signaling. We therefore propose that AHRi directly inhibits AHR and downregulates STAT1 leading to HLA-E downregulation.

Although AHR has been shown to be associated with monocytic AML maturation and a distinct genomic signature, the immune profile has not been fully explored. Utilizing the Beat AML dataset of 560 patients with RNAseq data, published by our group in collaboration with others, we ranked all *de-novo* samples by normalized *AHR* expression with “high” and “low” expression groups based on the 90th and 10th percentiles, respectively. Group 1 was labeled as “*AHR* high” and group 2 labeled as “*AHR* low”. Genes with increased expression in the *AHR* high samples were enriched in Reactome pathways such as TCR signaling, IL10 signaling, PD1 and IFNγ signaling. While genes with increased expression in the *AHR* low group were enriched in Reactome pathways pertaining to DNA repair and telomere maintenance (**Figure 3A**) (**Supplementary Figure 2A**) (51). In addition, when ranking *AHR* RNA levels by immune ligands of interest, the *AHR* low and high samples clustered into three groups of samples: Cluster1 and Cluster2 were mainly comprised of *AHR* low samples while *AHR* high samples were found in Cluster3. With respect to the AHR pathway genes and immune markers, Cluster1 had decreased expression, was (i.e., ‘immune cold’), Sample Cluster3 had increased expression, (i.e., ‘immune hot’), while Cluster2 had mid-range expression (**Figure 3B**). The *AHR* low group had the lowest expression genes encoding MHC class I and II as well as key immune markers, including downregulation of immune checkpoint molecules (*LILRB1, HLA-E, CD274* (*PD-L1*) and *IFNG*. In addition, *AHR* high AML samples had lower percentages of total CD56+ NK cells as well as lower mature NK cells defined as lineage negative (CD3^-^, CD14^-^, CD20^-^), CD56^dim^, or CD94^+^ CD16^+^ NK cells (**Supplementary Figure 2E**, **Figure 3C**). These findings suggest a connection between AHR signaling and the complex immune microenvironment that is critical for dictating immune response. Monocytic AML defined by high AHR may be less susceptible to immune based killing. Overall, our data suggests that monocytic AML is associated with higher AHR expression but also a unique immune profile which may be deleterious to overall survival in AML patients. To determine the baseline levels of IFN-γ in monocytic AML and effect of IFN-γ on leukemic blasts, we exposed MOLM-14 cells to IFN-γ and measured AHR levels by western blotting. We found that interferon gamma upregulated AHR after 24 hours in MOLM-14 cells (**Figure 3D**) mimicking the proposed changes we see in the microenvironment with NK or T cell IFN-γ secretion. To confirm this, we also performed genetic knockdown of *IFNGR1* in MOLM-14 and found that AHR secretion was dependent on the presence of *IFNGR1* likely due to a dependency on JAK/STAT signaling to produce AHR upregulation (**Supplementary Figures 2C, D**) and also described recently in another published manuscript (52). We therefore propose that AHRi mediated control of HLA-E is related to STAT1 signaling controlled by IFN-γ (**Figure 3E**). To validate the source of IFN-γ secretion in the microenvironment and to obtain more accurate NK cell characteristics including NK cell receptor/ligand pairs in AML patients, we performed single-cell RNA sequencing on *AHR* high vs *AHR* low peripheral blood samples.

### High AHR AML is associated with abnormal NK cell profiles in peripheral blood

**Figure 4:**
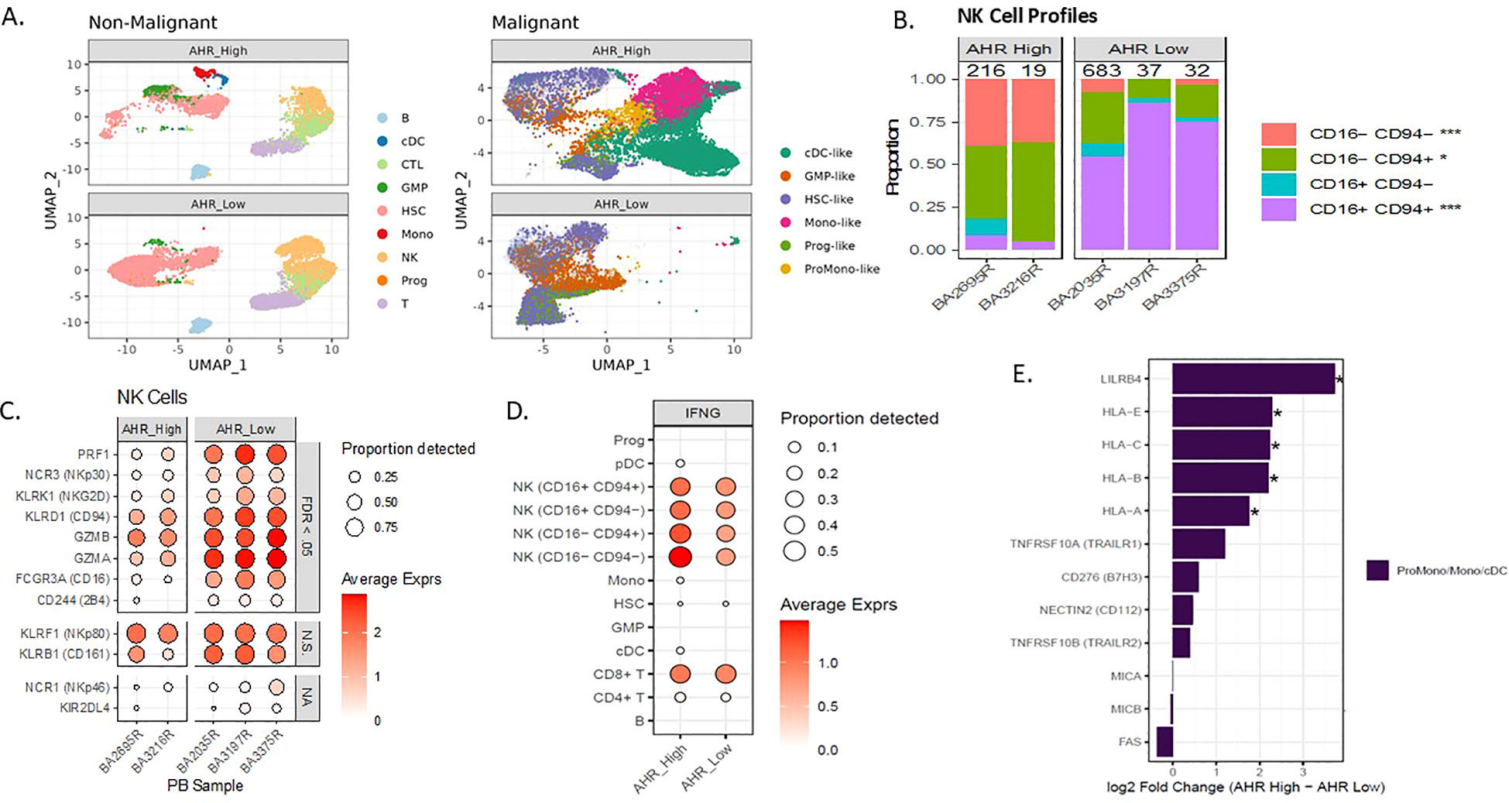
AHR pathway activation in AML correlates with abnormal NK cell profiles in PB. **(A)** Single cell UMAP clustering of AML peripheral blood samples with predicted non-malignant and malignant cell profiles separated by AHR high and AHR low samples. **(B)** NK cell maturation of single cell samples based on percentage of CD94/CD16 in 2 AHR high vs 3 AHR low (n=5). **(C)** Single cell NK cell receptor average expression (based on color scale) and proportion detected (size of circle) on AHR high and low samples. Significance indicated by FDR <0.05, N.S. indicates non-significance, NA indicates not evaluable. **(D)** Average expression and proportion detected of IFN-γ across the single-cell subsets for both AHR high and AHR low samples. **(E)** Pseudobulk differential expression of blast ligands on monocyte-like blasts in AHR high and AHR low samples. *FDR <.05.

AML evades both innate and adaptive immunity through several defined mechanisms and may be dependent on maturation state of the leukemic blast (53–55). The interplay between leukemic blast maturation state and immune recognition has not been clearly defined in the context of AHR. We previously looked at the role of NK cell percentage and number in newly diagnosed AML samples and found that higher NK cell percentage in the marrow was associated with worse OS. We therefore wanted to determine if NK cell profiles in the peripheral blood were different among monocytic AHR high AML samples vs low. We used single-cell RNAseq to characterize NK maturation and receptor-ligand pairs in 2 *AHR*-high (>8 RPKM) and 3 *AHR*-low (<5 RPKM) peripheral blood samples from AML patients. Characteristics of these patient samples are displayed in **Supplementary Table 4**. Cell type clusters in these samples are displayed using UMAP for AHR high and AHR low malignant cells and non-malignant cells to determine cell type differences (**Figure 4A**). Consistent with our flow cytometric analysis (**Figure 3C**), single cell analysis of the 5 AML samples demonstrated that *AHR*-high samples have a lower percentage of mature CD56^dim^, CD94^+/-^ CD16^+^ “stage 5” NK cells in the peripheral blood compared to *AHR*-low samples (**Figure 4B**). In addition, NK cells identified in AHR-high samples had less expression of NCRs (*NKp30*), *CD16, NKG2D*, and *2B4* (**Figure 4C**) (56). NK cells from *AHR*-high samples also had lower expression of perforin (*PRF1*) and granzyme A/B (*GZMA, GZMB*), suggesting that *AHR*-high NK cells are hypofunctional compared to *AHR*-low samples. Previous studies have shown that AHR directly binds to the promoter of *IFNG* in human NK cells; thus, we hypothesized that the NK cells are secreting IFN-γ rather than an autocrine process by leukemic blasts. We indeed found that IFN-γ was highest in NK cells (particularly the CD94-, CD16- subset, likely stage 2 immature NK cell) and cytotoxic CD8+ T lymphocytes and not found in leukemic blasts (**Figure 4D**). We also looked at T cell subsets, defined as CD3^+^ CD8^+^ or CD4^+^ cells and found no significant differences between *AHR* groups (**Supplementary Figure 3A**). To pair our immune findings with leukemic blast differentiation state, we examined NK receptor ligands (NKRLs) known to impact T and NK cell function (including MHC Class I, *LILRB4, TRAILR1, B7H3, CD112, TRAILR2, MICA/B* and *FAS*) based on differentiation state of the leukemic blast defined by single-cell sequencing (HSC/Prog state being more immature while the Promono/Monocytic/Dendritic cell leukemic blast being more mature), as previously described (29, 37). Among the NKRLs examined, NKG2D receptor ligands (*MICA, MICB ULBP1, ULBP2/5/6*) and *FAS* are known to confer an activating signal to NK cells while MHC class I, *LILRB4, B7H3, TRAIL1, PDL1*, and *PDL2* are known to inhibit NK cell function (5, 57). *AHR*-high samples had significantly higher expression of NK cell inhibitory receptors MHC Class I (*HLA-A, -B, -C, -E*) and *LILRB4* (FDR <.05) than *AHR*-low samples on monocytic leukemic blasts (**Figure 4E**). MHC Class I subsets among all leukemic blast maturation states are shown in **Supplementary Figure 3B**. *CD112, TRAILR1*, and *B7H3* also trended higher in *AHR*-high samples. Absence of NKG2D ligands on stem cells were previously reported to be a mediator of immune evasion (7). Interestingly, MHC Class I was more highly expressed on both HSC and mature leukemic blasts in the *AHR*-high samples compared to *AHR*-low (**Supplementary Figure 3C**). In summary, our data supports other published reports linking differentiation state of the leukemic blast with immune sensitivity, which may be driven by AHR (5, 7, 37). Given the relatively high transcript levels for MHC Class I and *HLA-E* and the lower expression of NK cell activating receptors in *AHR*-high samples, we hypothesized that blocking the AHR pathway with an AHR inhibitor may enhance NK cell killing through modulating leukemic blast ligands.

### AHR inhibitor enhances NK cell cytotoxicity and downregulates HLA-E and STAT1

**Figure 5:**
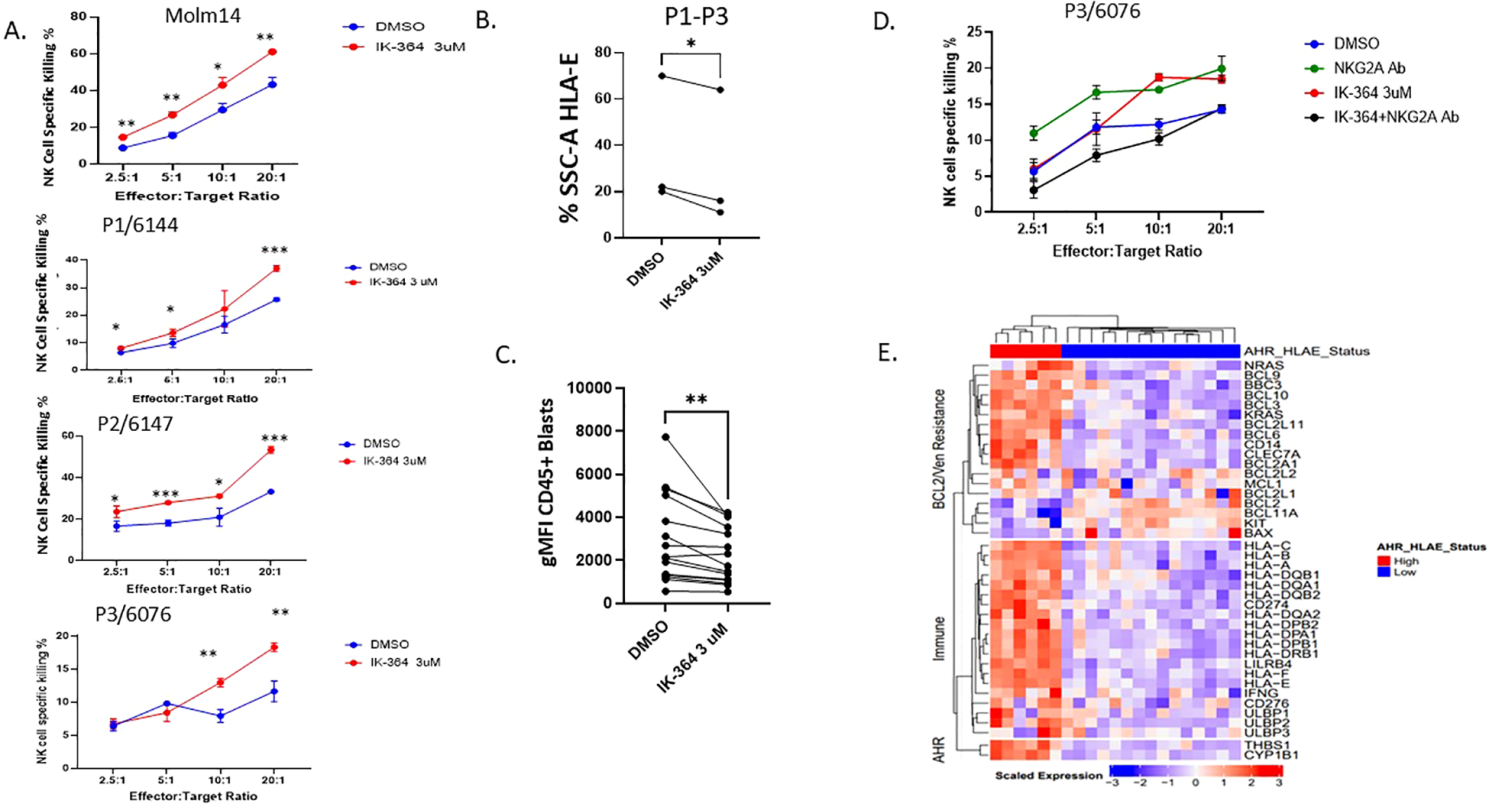
AHR inhibitor, IK-364, enhances NK cell cytotoxicity and downregulates HLA-E. **(A)** NK cell killing assay on MOLM-14 cell line (repeated in triplicate) and patient samples pre-exposed to IK-364 3 µM for 48 hours prior to NK co-culture. Each dot has 3 replicates with error bars shown. p<0.05, ** p<0.01, ***p<0.001 **(B)** HLA-E protein expression based on % SSC-A of live cell HLA-E + by flow cytometry between vehicle treated (DMSO) or IK-364 for 48 hours in patient samples (P1-P3), paired student T test P<0.05. **(C)** HLA-E geometric mean florescent intensity (gMFI) between DMSO or IK-264 treated AML patient samples (n=12). ** P<0.001 **(D)** Primary patient sample (P3) with antecedent CMML/AML treated with NKG2A blocking antibody 1-hour prior NK cell exposure. **(E)** Heatmap of immune and Venetoclax resistant marker RNA expression in beat AML for patients with dual expression of AHR and HLA-E (high/high and low/low; n=21).

HLA-E is the major ligand for the inhibitory receptor CD94/NKG2A found on both NK cells and subsets of cytotoxic CD8^+^ T cells and is highly conserved among MHC Class I genes (48). The expression and role of HLA-E is not well-characterized in AML; however, downregulation can lead to enhanced NK cell recognition. IK-364 is a potent AHR inhibitor in pre-clinical development. Dose-finding studies showed that IK-364 is a potent inhibitor of AHR, demonstrated by the decreased expression of AHR downstream target CYP1B1 at all tested (**Supplementary Figure 1C**) and noted to have an IC_50_ of 5nM in MOLM-14 cells. Of note, the 3µM IK-364 dose was not associated with direct cell apoptosis (**Supplementary Figure 1E**). To determine the functional impact of blocking the AHR pathway on leukemic blasts, MOLM-14 cells were cultured with IK-364 3uM for 48 hours. Compared with the vehicle control group, pre-treatment with 3 µM IK-364 enhanced NK cell-mediated cytotoxicity against MOLM-14 as well as in primary patient samples (**Figure 5A**) of different FAB classification subsets (**Supplementary Figure 5**). Clinical characteristics of all patients are shown in **Supplementary Table 4**. Furthermore, this enhanced NK cell killing after exposure to IK-364 also correlated with a downregulation of HLA-E in patient samples (**Figure 5B**) and in 12 patient samples (**Figure 5C**). To validate the significance of HLA-E as mediator of enhanced NK cell killing, we performed assays using NK cells treated with a NKG2A blocking antibody for 30 minutes prior to adding them to the targets. We found that the addition of the NKG2A blocking antibody blocked the effect of IK-364 in a patient sample with high HLA-E, suggesting that the HLA-E-NKG2A axis is a major driver of NK cell resistance (**Figure 5D**). To determine the mechanism behind HLA-E downregulation with IK-364, we performed bulk RNAseq sequencing to assess expression of gene encoding transcription factors known to regulate HLA-E such as GATA2, CIITA, STAT1, IRF1, NF-kB and HOXA5. We found significant downregulation of both STAT1 (FDR p=0.012) and IRF1 (FDR p=0.016) when exposed to 24 hours of 3uM IK-364 compared to DMSO alone (**Supplementary Figure 6**). Kynurenine, STAT1 and IRF1 are closely connected through the IFN-y signaling pathway. IFN-γ activates STAT1 leading to induction of IRF1. IRF1 binds to the promoter of the IDO1 gene, which is the enzyme that catalyzes the conversion of tryptophan to kynurenine initiating the kynurenine/AHR pathway. To determine if AHR mediates direct regulation of HLA-E (outside of STAT1/IFN-γ)we performed genetic KO of AHR and did not see a significant impact on HLA-E expression (**Supplementary Figures 7A, B**) suggesting that HLA-E regulation by AHR is likely through an indirect mechanism (ie. IRF1 and/or STAT1) rather than direct. Given the connection between monocytic AML and BCL2 inhibitor resistance, we set out to determine if the co-expression of AHR and HLA-E could discriminate BCL2 family expression as well as expression of an immune repertoire. To our surprise, the co-expression of both HLA-E and AHR by RNA, clearly separated patients visually into immune “hot”/high AHR states vs immune “cold”/AHR low states (**Figure 5E**) where “hot” indicates higher relative centered/scaled expression (Zscore > 0) and “low” indicates lower relative expression (Zscore < 0). These data add to the growing literature of a dual role of AHR signaling in controlling key mediators of immune regulation as well as leukemic blast cell differentiation state. Our work highlights a continued interest in the role of AHR blockade in AML and adds to the literature on how AHR inhibition may enhance NK cell killing through modulating the expression of leukemic blast ligands that are critical for NK cell function.

## Discussion

Herein we show that higher NK cell percentage at diagnosis in the bone marrow negatively impacts OS in newly diagnosed AML patients treated with standard induction intensive 7 + 3 chemotherapy. We found that these patients had higher relative expression of HLA-E on leukemic blasts and higher CD94+ NK cells. Importantly, we found that higher T cell numbers were associated with improved OS in this cohort as well as a correlation between low NK cell % patients having higher MHC Class II expression. Through the Beat AML dataset, we were able to correlate HLA-E expression with AHR and monocytic maturation. We further define AHR as a surrogate marker for immune dysregulation with lower percentage of mature NK cells found in the peripheral blood by flow cytometry and single cell sequencing. Unique to our work, we identified an immune signature associated with AHR, defined by high levels of HLA Class I, HLA-E and IFN-γ signaling, which may impact treatment response to cellular therapy. We show *in-vitro* that IFN-γ exposure upregulates AHR in leukemic blasts after just 24 hours mimicking the role of T and NK cell in the microenvironment. NK cells from *AHR*-high patients had impaired maturation profiles characterized by less mature CD16+/CD56dim NK cells and had less activating NCRs including *NKp30, CD16, 2B4* and *NKG2D*. When primary patient samples were exposed to the AHR inhibitor, IK-364, HLA-E protein was downregulated on leukemic blasts and NK cell cytotoxicity was enhanced. Lastly, we found that IK-364 downregulated STAT1 and IRF1 in MOLM-14 cells and propose STAT1 as the major mediator connecting AHR inhibition to downregulation of HLA-E.

AML has many mechanisms for evading innate and adaptive immune control critical for predicting response to immune based therapies. NK cells isolated from AML patients have decreased expression of NCRs NKp46, NKp30, NKp44, NKp80, and 2B4, increased expression of inhibitory CD94/NKG2A, shedding of activating ligands (e.g., CD16) and decreased killing activity (58). AML cells can also stimulate immunosuppressive effects on NK cells by supporting regulatory T cells (Tregs) and inhibiting dendritic cell function. Wang et al. recently showed the significance of IFN-γ signaling in venetoclax resistance and paired immune markers including MHC Class I and HLA-E expression (59). Although they showed convincing evidence of the role of immune suppression in monocytic AML, their report lacked functional studies and did not address mechanisms to reverse this defect for clinical applications. We largely validated their finding by showing that monocytic AML was highly correlated with HLA-E expression, as well as linking the monocytic driver, AHR, to these immune changes and IFN-γ through bulk and single cell sequencing. Our work highlights the AHR -IFN-γ cross talk, which may be a driver of immune evasion and HLA-E in AML. Our findings also support and validate that NK cell maturation is altered in AML patients relative to AHR expression in the peripheral blood. In particular, previous publications have shown that AHR activation leads to expansion of type 1 innate lymphoid cells (ILC1), a NK cell maturation block and fewer NK cells (1, 27, 28). Our current work adds to this literature by linking AHR as a potential targetable pathway to enhance NK cell therapy in AML.

We had several limitations in our study which could impact our findings. First, we did not account for a newly described AML subset “CD56 neg CD16+” NK cells that were recently found to be associated adverse clinical outcome in AML (60). In the manuscript, AML patients with expanded CD56neg CD16+ NK cells group had significantly poorer overall survival (HR[CI95]=3.3[0.75-14.7], p=0.0251) and relapse-free survival (HR[CI95]=13.1[1.9-87.5], p=0.0079) after 36 months follow-up (60). It is possible that this NK cell subset contributed to our differences in OS however was not accounted for in our analysis. Second, we were not able to connect our initial bone marrow findings of NK cell percentage in the marrow with AHR levels or FAB maturation state despite RNA sequencing overlap between our cohorts. We believe the reason for this may be related to the relative expression differences of AHR in bone marrow vs peripheral blood, since AHR levels in the bone marrow are much lower than in the peripheral blood. Previous publications have shown that AHR signaling is preferentially downregulated in LSC-enriched populations within the bone marrow to allow for stem cell maintenance (25). Another limitation to our study is the small sample size for single cell data analysis in the peripheral blood samples and not having paired bone marrow samples. In addition, we showed a single patient sample validating NKG2A as a major driver of NK cell dysfunction in a high HLA-E expressing patient. However, these results are still reportable given the current validated work in the field and our correlation with the larger Beat AML dataset confirming HLA-E presence in AHR high samples, MHC class I/II and NK cell receptor-ligand differences. Lastly, we did not show how AHR directly downregulates HLA-E in AML. Previous literature suggests that it is medicated through interferon gamma crosstalk rather than AHR directly binding to the HLA-E promoter. Our experiments with genetic knock out of AHR did not change relative protein expression of HLA-E in the KO cell line compared to NT suggesting the mechanism is not direct. Genetic knockdown of IFNGR1 on MOLM-14 cells prevents AHR secretion by IFN-γ, in line with a prior publication by Snyder et al. which links AHR to IFN-γ-induced JAK/STAT pathway and immune checkpoint-mediated immunosuppression in lung adenocarcinoma (52). Based on our current understanding and available data, we believe IFN-γ triggers a signaling cascade involving the IFN-γ receptor, Janus kinases (JAKs), and ultimately leads to the phosphorylation and activation of STAT1. STAT1 then can bind to the HLA-E gene promoter, increasing HLA-E transcription and expression (61). Activation of the JAK/STAT pathway leads to the transcription of IRF1, and IRF1, in turn, can contribute to a positive feedback loop by promoting STAT1 phosphorylation and DNA binding, further enhancing the JAK/STAT signaling (62). We therefore propose that AHRi directly inhibits AHR and downregulates STAT1 causing HLA-E downregulation (**Figure 3E**). We acknowledge that AHR may be modulating several other ligands on leukemic blasts other than HLA-E. We suspect that the proinflammatory environment characterized by high IFN-γ levels and other microenvironmental factors are also important mediators of NK cell resistance. Although we highlight MHC Class I, including HLA-E, we acknowledge that there may be multiple AHR-induced changes on leukemic blasts (e.g. MHC Class I, MHC Class II, LILRB4) that contribute to impaired NK cell cytotoxicity. Future experiments will investigate these multifactorial changes in immune ligands vs maturation state via genome-wide CRISPR screens with NK cells to determine and validate the targets most critical for augmenting NK cell function. In addition, future studies will work to clarify the role of monocytic/venetoclax resistant AML and the role for immune modulation in the setting of cellular therapy.

In summary, our work highlights the immune profiles associated with AHR in AML and supports continued interest in targeting the AHR pathway to augment immune-based therapies in AML.

## Data Availability

All sequencing data, along with relevant clinical annotations have been submitted to dbGaP and Genomic Data Commons (GDC) and are publicly available. The dbGaP study ID is 30641 and accession ID is phs001657.v2p1 (https://www.ncbi.nlm.nih.gov/projects/gap/cgi-bin/study.cgi?study_id=phs001657.v2.p1). The single-cell data are available in the GDC as part of the BEATAML1.0-COHORT project.
