# Supplementary material for: The aryl hydrocarbon receptor is associated with monocytic AML and innate immune resistance reversible with an AHR inhibitor
Source: Front Immunol. 2025 Dec 9;16:1554166. doi: 10.3389/fimmu.2025.1554166 (PMC12722994; doi:10.3389/fimmu.2025.1554166)

Supplementary Information

Supplementary Table 1. Patient Characteristics of 69 newly diagnosed AML patients treated with 7+3 induction, by median-dichotomized NK cell % (of lymphocytes) group.

Characteristic	Low NK cell % (n=35)	High NK cell % (n=34)	p-value
**AML Type**			0.614
**De novo**	n = 34 (97%)	n = 32 (94%)	
**Secondary**	n = 1 (3%)	n = 2 (6%)	
**Age**			0.015
**Median (IQR)**	53 (40 - 62)	62 (53 - 67)	
**Range**	18-70	24-83	
**Biological Sex**			0.338
**Female**	n = 19 (54%)	n = 14 (41%)	
**Male**	n = 16 (46%)	n = 20 (59%)	
**ELN Risk**			0.088
**Favorable**	n = 19 (54%)	n = 10 (29%)	
**Intermediate**	n = 6 (17%)	n = 12 (35%)	
**Adverse**	n = 10 (29%)	n = 12 (35%)	
**FLT3-ITD mutation**			0.392
**No**	n = 28 (80%)	n = 24 (71%)	
**Yes**	n = 6 (17%)	n = 10 (29%)	
**NA**	n = 1 (3%)	n = 0 (0%)	
**TP53 mutation**			1.000
**No**	n = 31 (89%)	n = 30(88%)	
**Yes**	n = 3 (9%)	n = 4 (12%)	
**NA**	n = 1 (3%)	n = 0 (0%)	
**IHD1/2 mutation**			1.000
**No**	n = 30 (86%)	n = 29(85%)	
**Yes**	n = 4 (11%)	n = 5 (15%)	
**NA**	n = 1 (3%)	n = 0 (0%)	
**FAB Class**			
**M0**	n = 1 (3%)	n = 4 (12%)	
**M1**	n = 3 (9%)	n = 8 (24%)	
**M2**	n = 13 (37%)	n = 12(35%)	
**M4**	n = 13 (37%)	n = 6 (18%)	
**M5**	n = 3 (9%)	n = 3 (9%)	
**NA**	n = 2 (6%)	n = 1 (3%)	
**WBC (10^3/μl)**			0.914
**Median (IQR)**	16.0 (6.5 - 35.5)	13.5 (4.0 - 49.8)	
**Range**	0.5 - 198.0	1.0 - 138.0	

**Blast % (of WBC)**<br>**Median (IQR)**<br>**Range**	 51.6 (31.0 - 68.1)<br>6.7 - 95.1	 61.4 (35.0 - 81.8)<br>13.5 - 95.6	0.220
**Lymphocyte % (of WBC)**<br>**Median (IQR)**<br>**Range**	 10.2 (4.7 - 18.2)<br>2.2 - 41.0	 7.3 (4.4 - 16.6)<br>1.4 - 53.6	0.734
**T cell % (of lymphocytes)**<br>**Median (IQR)**<br>**Range**	 72.9 (61.7-79.8)<br>43.8 - 90.9	 72.2 (58.5 - 78.6)<br>41.5 - 85.0	0.563
**T cell number (per µL)**<br>**Median (IQR)**<br>**Range**	 1026 (641 - 2077)<br>57 - 3482	 1001 (495 - 1698)<br>107 - 7050	0.463
**NK cell % (of lymphocytes)**<br>**Median (IQR)**<br>**Range**	 5.7 (3.8 - 6.9)<br>2.1 - 8.7	 13.0 (10.2 - 14.7)<br>8.8 - 22.6	0.000
**NK cell number (per µL)**<br>**Median (IQR)**<br>**Range**	 86.0 (57.1 - 111.7)<br>4.9 - 376.5	 166.9 (88.8 - 308.0)<br>32.1 - 1321.9	0.002
**Response to induction**<br>**CR/CRi**<br>**PR**<br>**None**	 n = 31 (89%)<br>n = 0 (0%)<br>n = 4 (11%)	 n = 26 (76%)<br>n = 0 (0%)<br>n = 8 (24%)	0.218

Supplementary Figure 1A. Chemical Structure of IK-364 (IKENA Oncology). B. Dose Response Curve for IK-364 in MOLM14 cell line from 0-10000 nM. C. CYP1B1 PCR fold change in MOLM-14 cell line exposed 72 hours to IK-364 at various dose levels for 72 hours.

A.

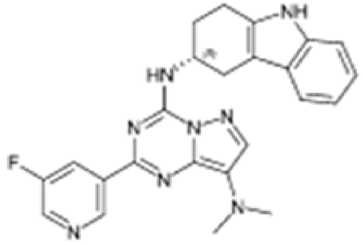

B.

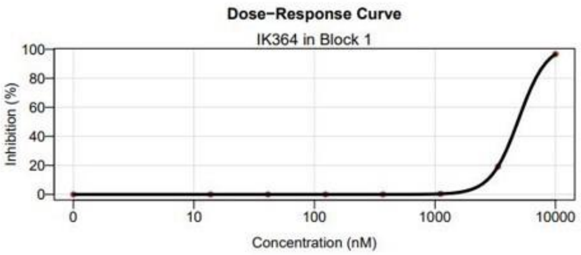

C.

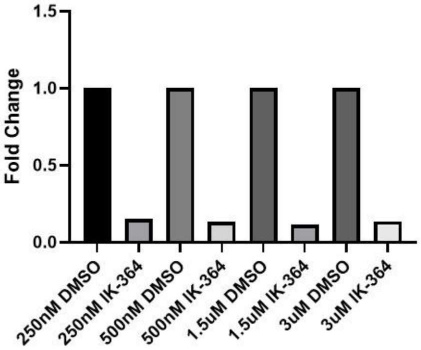

Supplementary Figure 1D. Differential gene expressions were performed on bulk RNAseq data from 20 ND AML patient samples (in Beat AML) with ether “low” (n=10) or “high” (n=10) NK cell percentages (of lymphocytes). Note this NK cell% low vs high RNAseq patient cohort is a subset of the N=69 patient clinical cohort for whom OS is analyzed. Global genewise modeling of expression differences between NK cell% low and high groups controlled for the sequenced sample type and was performed using surrogate variable analysis (to identify unobserved covariates for the model) and the limma-voom procedure.

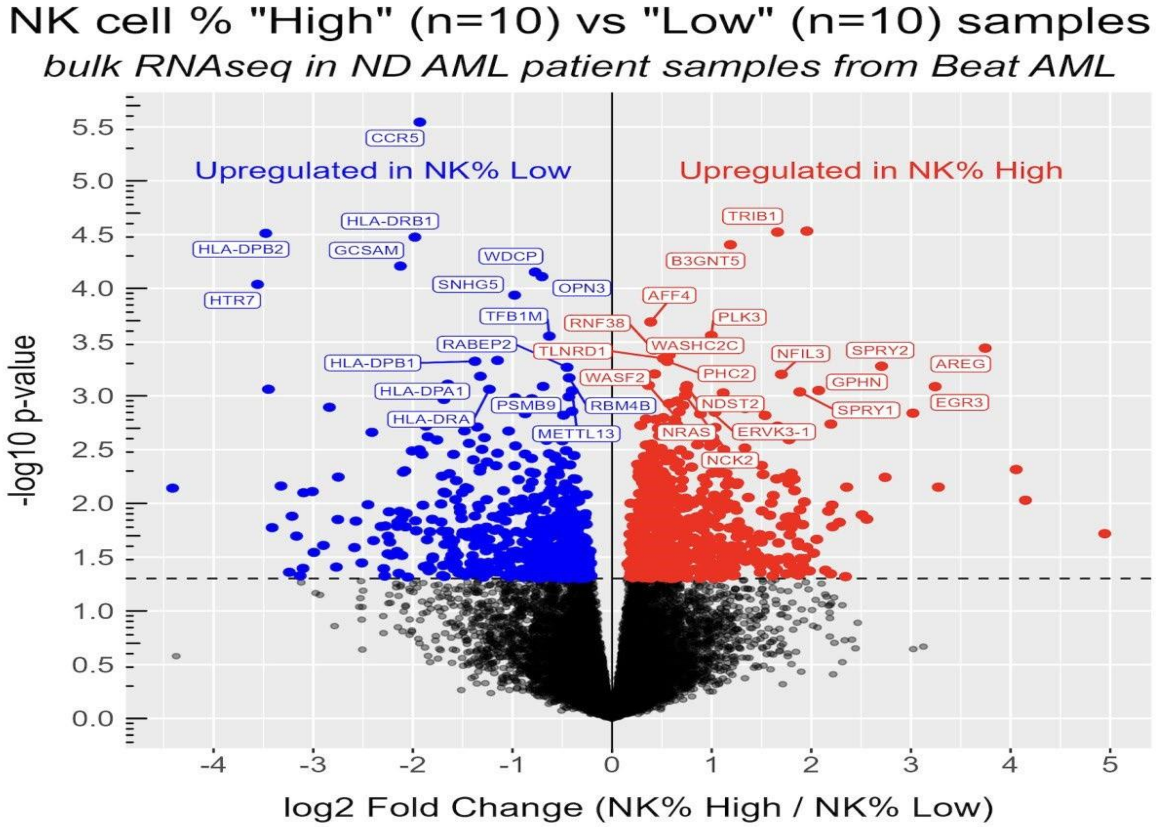

Supplementary Figure 1E. Healthy human and AML patient samples Peripheral Blood Mononuclear Cells (PBMC) co-cultured with DMSO, 500 nM IK-364 or 3uM IK-364 for 24 hours and measured for annexin stain. All samples were frozen PBMCs.

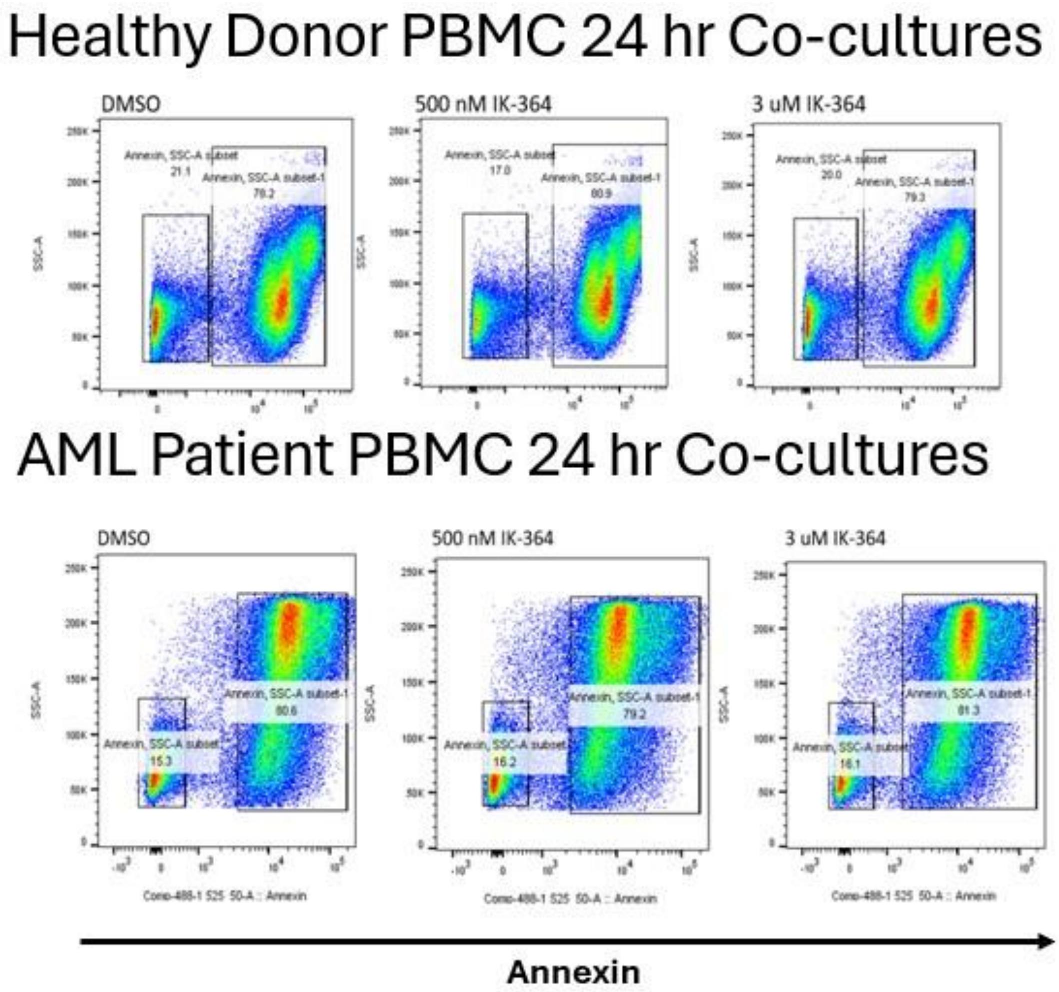

Supplementary Figure 1F. NK cell killing assay with two different concentrations of IK-362 500nM and 3uM with MOLM14 cells.

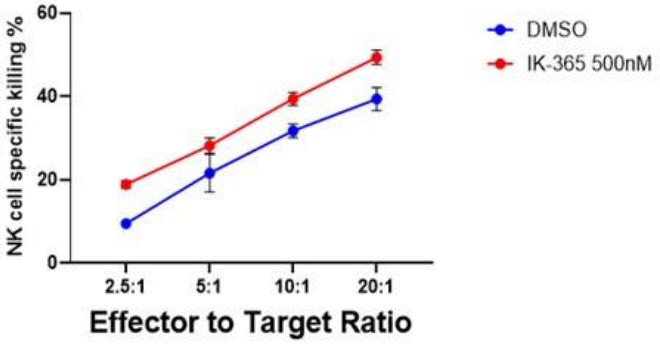

Supplementary Figure 2A. Reactome pathway analysis of top 10% AHR based on RPKM and shows pathway activation in top 10% of AHR high samples.

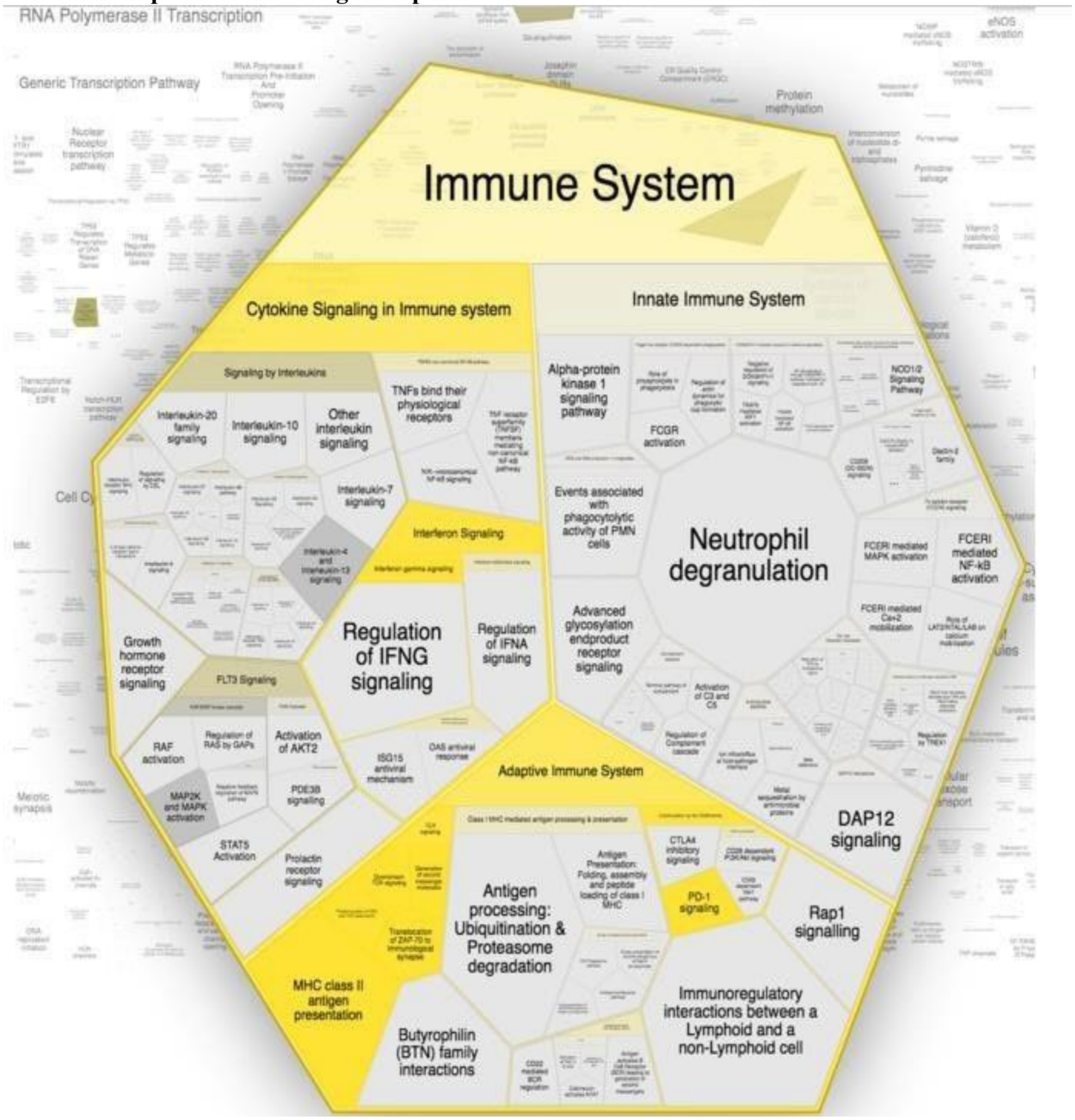

Supplementary Figure 2B. IFNGR1 expression among FAB subtypes in the Beat AML dataset.

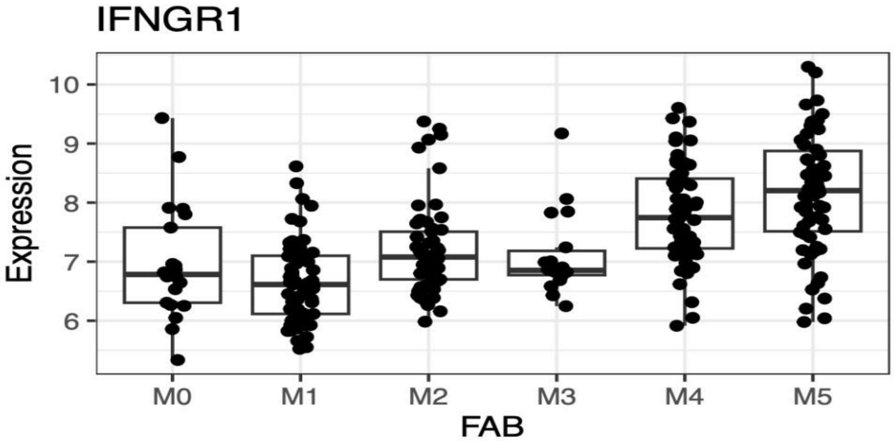

Supplementary Figure 2C. Genetic knockdown of IFNGR1 in MOLM-14 AML and interferon gamma stimulation of AHR for 24 hours.

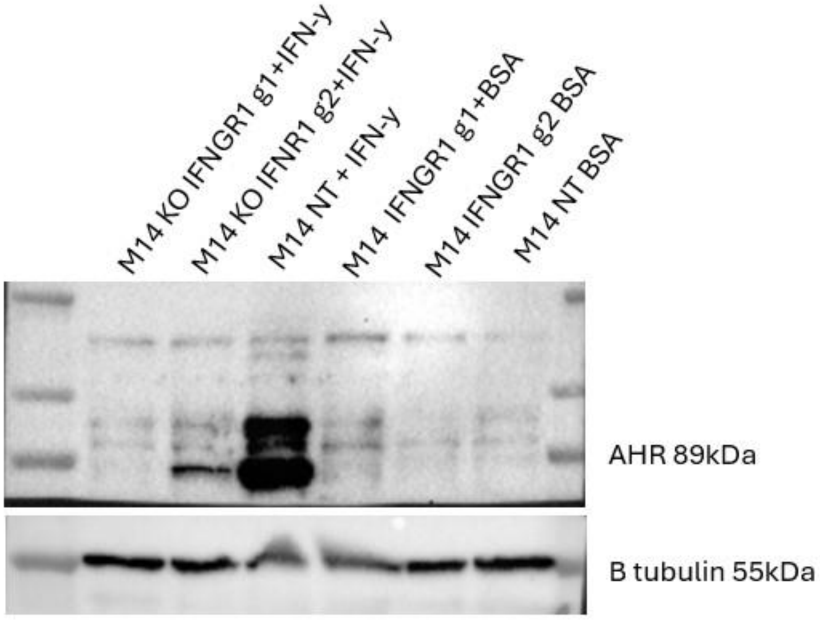

Supplementary Figure 2D. Expression of IFNGR1 (CD119) on MOLM-14 cell lines with IFNGR1 g1 and IFNGR1 g2 and non-targeting (NT). Bovine Serum Albumin (BSA) control.

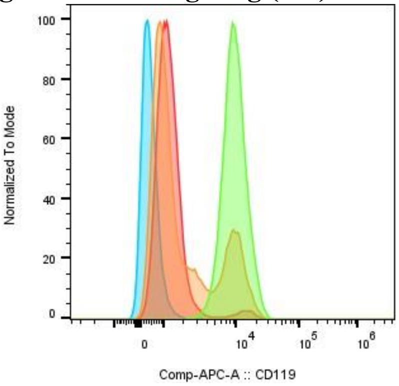

Sample Name	Subset Name	Count	Geometric Mean : Comp-APC-A
M14 sgNT BSA.fcs	Comp-Zombie Aqua-A, SSC-H subset	94300	9122
M14 sgIFN-y_2 BSA.fcs	Comp-Zombie Aqua-A, SSC-H subset	94363	1972
M14 sgIFN-y_1 BSA.fcs	Comp-Zombie Aqua-A, SSC-H subset	94280	1317
Unstained.fcs	Comp-Zombie Aqua-A, SSC-H subset	93662	448

Supplementary Figure 2E. Flow cytometry summary statistics showing 11 patient samples which are AHR high or AHR low (n=11) and relative % of NK cells of lymphocytes based on AHR high (n=5) or low (n=6) on lineage negative CD56+ cells (n=11).

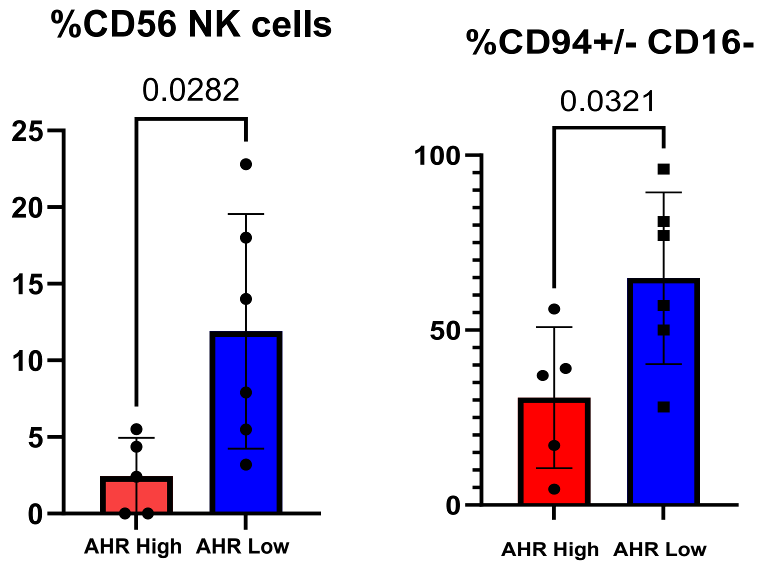

Supplementary Figure 3A. Single cell T cell profiles in 5 primary AML samples.

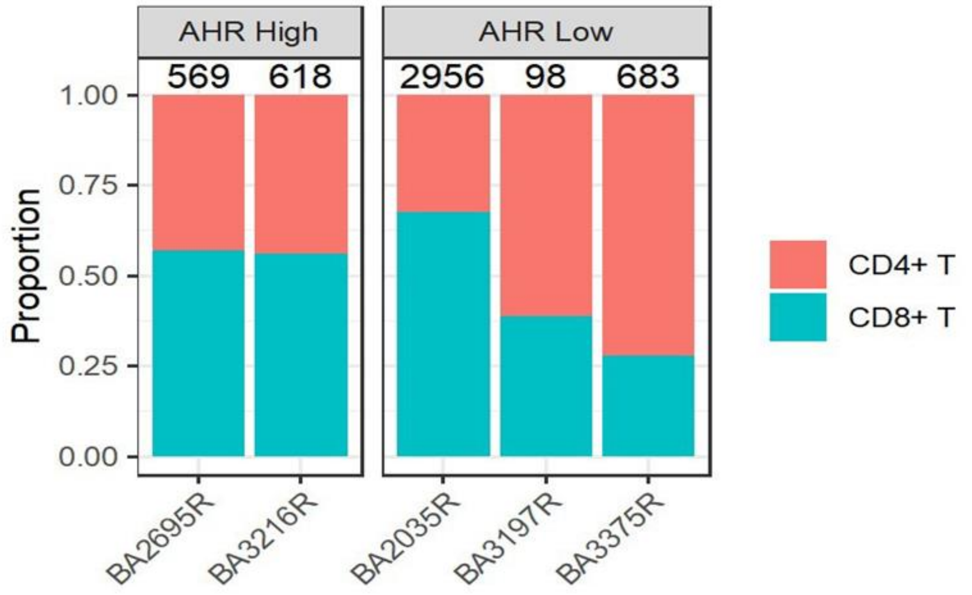

Supplementary Figure 3B. MHC Class I expression on different blast subsets based on pseudobulk differential expression between samples with AHR high and AHR low expression. *FDR < .05.

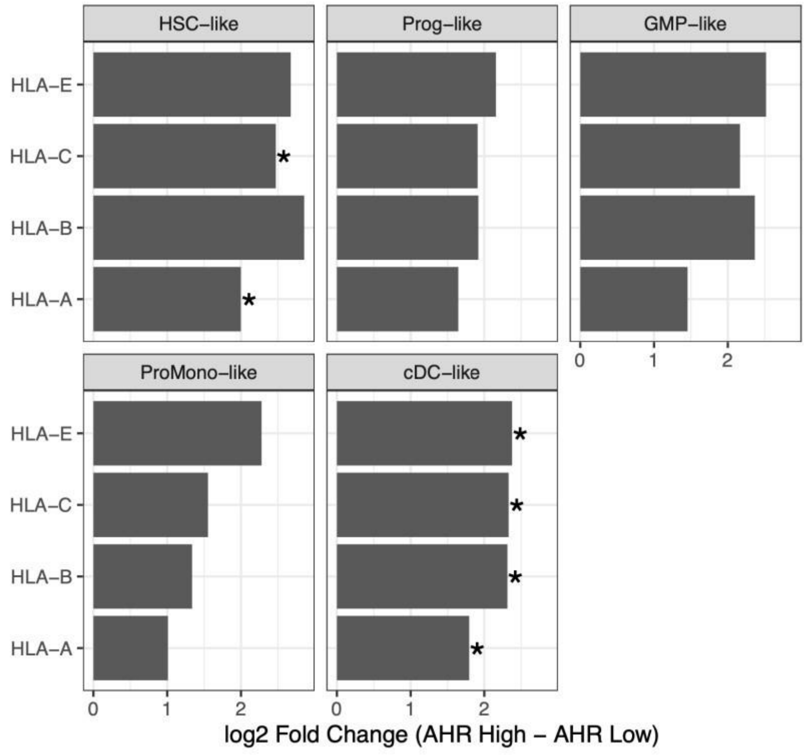

Supplementary Figure 3C. Plot of pseudobulk differential expression log2 fold change relative to the HSC/Prog vs ProMono/Mono/cDC blasts differentiation states for NK cell ligands among peripheral blood single cell AML patient samples. *FDR < .05

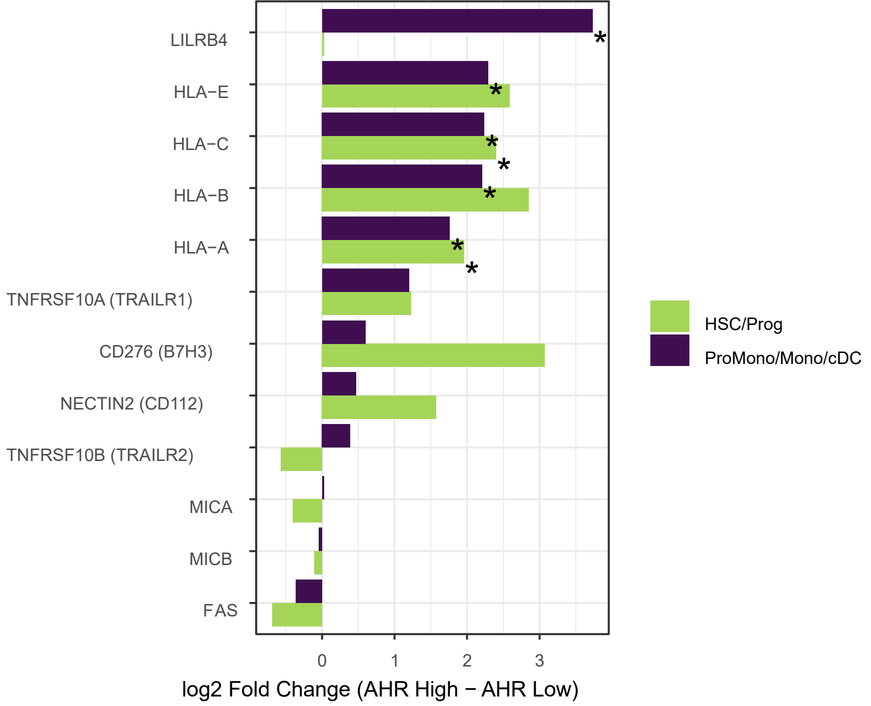

Supplementary Table 2. List of antibodies used for flow cytometry.

Antigen	Clone	Color	Source
**CD3**	UCHT1	BV 785	Biolegend
**CD14**	HCD14	PerCPCy55	Biolegend
**CD20**	RT20	VioGreen	Miltenyi
**CD34**	AC136	VioBlue	Miltenyi
**CD45**	HI30	BV 650	Biolegend
**CD56**	N901	APC-AF 700	Beckman coulter
**C94**	HP-3B1	PE vio 770	Beckman coulter
**CD13**	WM15	BV711	Biolegend
**CD16**	3G8	APC CY7	Biolegend
**HLA-E**	REA1031	PE-Vio 615	Miltenyi
**CD155**	SKIL4	PE	Biolegend

**LILRB4**	ZMU.1	AF647	Biolegend
**CD11b**	MI/70	APC	Biolegend

Supplementary Table 3. List of primer sequences for RT-PCR.

	Primer direction	Primer name	Primer Sequence
1	F	gapdhF	TGCACCACCAACTGCTTAGC
2	R	gapdhR	GGCATGGACTGTGGTCATGAG
3	F	Cyp1b1	ACGTACCGGCCACTATCACT
4	R	Cyp1b1	CTCGAGTCTGCACATCAGGA

Supplementary Table 4. Patient samples FAB, specimen type, disease characteristics and AHR levels based on Reads Per Kilobase of transcript per Million mapped reads (RPKM).

Sample ID	Group	FAB Classification *	Specimen Type	Disease Characteristics	Included in Single Cell
BA2201R	AHR High (FLT3+) >8.2	M5	Bone Marrow Aspirate	AML, karyotype normal. NPM1 p.W288fs* VAF 49% and FLT3-ITD VAF 90%	yes
BA2910R	AHR High (FLT3+) >8.2	M4	Peripheral Blood	AML with NPM1 and FLT3-ITD mutation (VAF unknown)	no
BA2455R	AHR High (FLT3+) >8.2	M5	Peripheral Blood	AML with NPM1 and FLT3 mutation (VAF unknown)	no
BA2935R	AHR High (FLT3+) >8.2	M5	Peripheral Blood	AML with NPM1 and FLT3 mutation (VAF unknown)	no
BA2762R	AHR High (FLT3+) >8.2	M2	Peripheral Blood	AML with NPM1 and FLT3-ITD mutation (VAF unknown)	no
BA2303R	AHR High (FLT3+) >8.2	M5	Peripheral Blood	AML with NPM1 and FLT3 mutation (VAF unknown)	no
BA2695R	AHR High (FLT3+) >8.2	M4	Peripheral Blood	Therapy related AML, FLT3 positive	yes
BA3216R	AHR High (FLT3+) >8.2	N/A	Peripheral Blood	AML NOS, FLT3 positive	yes
BA2374R	AHR Low (FLT3+) <6.8	M3	Bone Marrow Aspirate	Acute Promyelocytic Leukemia	no
BA2171R	AHR Low (FLT3+) <6.8	M1	Bone Marrow Aspirate	AML, Karyotype normal. FLT3-ITD VAF 46%, NPM1 p.W288fs*12 VAF 40%, IDH2 p.R140Q VAF 40%	yes
BA2501R	AHR Low (FLT3+) <6.8	M3	Peripheral Blood	Acute Promyelocytic Leukemia	no

BA2721R	AHR Low (FLT3+) <6.8	M2	Peripheral Blood	AML, Karyotype normal, FLT3-ITD VAF 47%, SF3B1 VAF 51%	no
BA2552R	AHR Low (FLT3+) <6.8	N/A	Peripheral Blood	AML, NOS, FLT3 positive	no
BA2776R	AHR Low (FLT3+) <6.8	M1	Peripheral Blood	AML, karyotype normal. FLT3-ITD VAF 70%	no

BA3036R	AHR Low (FLT3+) <6.8	N/A	Peripheral Blood	AML NOS, FLT3 positive	no
BA2035R	AHR Low (FLT3+) <6.8	M1	Peripheral Blood	AML, karyotype normal. FLT3-ITD VAF 40%, DNMT3A p.R882H VAF 50%	yes
BA3197R	AHR Low (FLT3+) <6.8	M1	Peripheral Blood	AML, FLT3 VAF 50%, NPM1 VAF 50%, IDH1 p.R132G VAF 47%	yes
BA3375R	AHR Low (FLT3+) <6.8	M1	Peripheral Blood	AML Karyotype normal. NPM1 VAF 48%, FLT3-ITD CAF 46%, IDH1 p.R132S VAF 47%	yes
6144 (P1)	N/A	CMML-2	Bone Marrow Sample	CMML-2, myeloid sarcoma. Karyotype normal. FLT3 p.D835Y VAF 22%, FLT3 p.N676K VAF 6%	no
6147 (P2)	N/A	M1	Bone Marrow Sample	AML, Karyotype normal. NPM1 p.W288fs*12 VAF 46%	no
6076 (P3)	N/A	M5	Bone Marrow Sample	CMML, transformed to AML, s/p alloHSCT on with relapse. Complex karyotype, NF1 p.C1817fs*52 VAF 99%	no

* Patients were considered to have monocytic AML, FAB M4 or M5 classification if they either had (1) morphology consistent with M4 or M5 by WHO 5th edition guidelines as assessed by an experienced clinical hematopathologist, or (2) if morphology was not available, had a minimum of at least three positive monocytic markers (CD11b, CD11c, CD14, CD64, CD36, and CD38) present on immunophenotypic analysis of a diagnostic AML specimen. N/A=missing data

Supplementary information on TCGA comparison

TCGA-related methods: Clinical data and expression (GAF2.0 normalized RPKM) for TCGA-LAML was downloaded from the Genomic Data Commons (https://gdc.cancer.gov/aboutdata/publications/laml_2012). Fusions were derived from reported gene fusion calls. Due to discrepancies between gene fusion calls and karyotypes, normal karyotype patients were identified as those who had a normal karyotype and no reported gene fusion. Gene expression was log2 transformed after adding 1.0. Monoctyte like signature was defined as previously published (DOI: 10.1016/j.ccell.2022.07.002). AHR thresholding was done using the top and bottom 10% of TCGA. Note the two fusions with fewer than five cases are indicated as N/A.

Supplementary Figure 4A. HLA-E vs fusion and corresponding Welchs T-stats (tcga_hlae_fig_stats.xlsx). Note the two fusions with fewer than five cases are indicated as N/A.

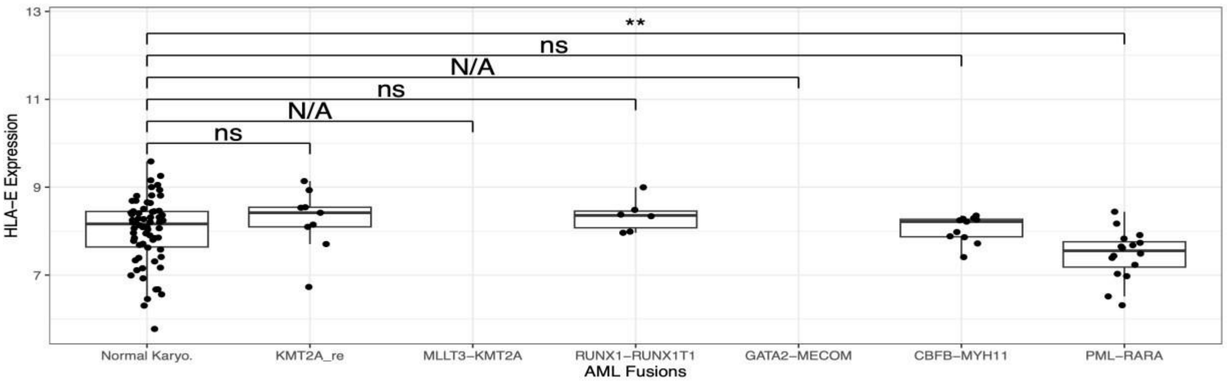

Supplementary Figure 4B. TCGA monocyte-like vs HLA-E (r=0.517; 0.4-0.617 95% CI; Pval: 1.31e-13; Pearsons correlation).

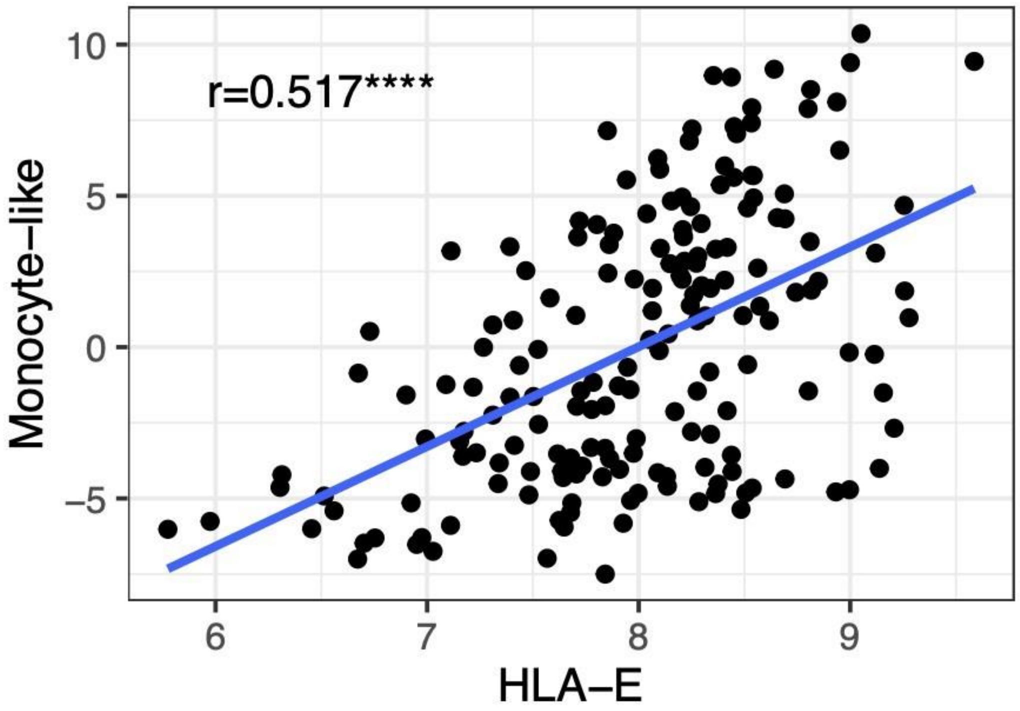

Supplementary Figure 4C. TCGA HLA-E vs AHR high vs low (High vs Low: 0.69; 0.272-1.11 95% CI; Pval: 0.00207; Welchs T-test).

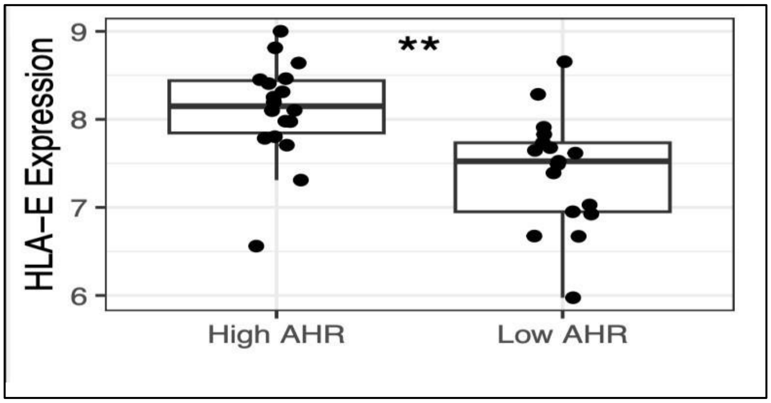

Supplementary Figure 5. NK cell killing assays on AML patients based on FAB classification.

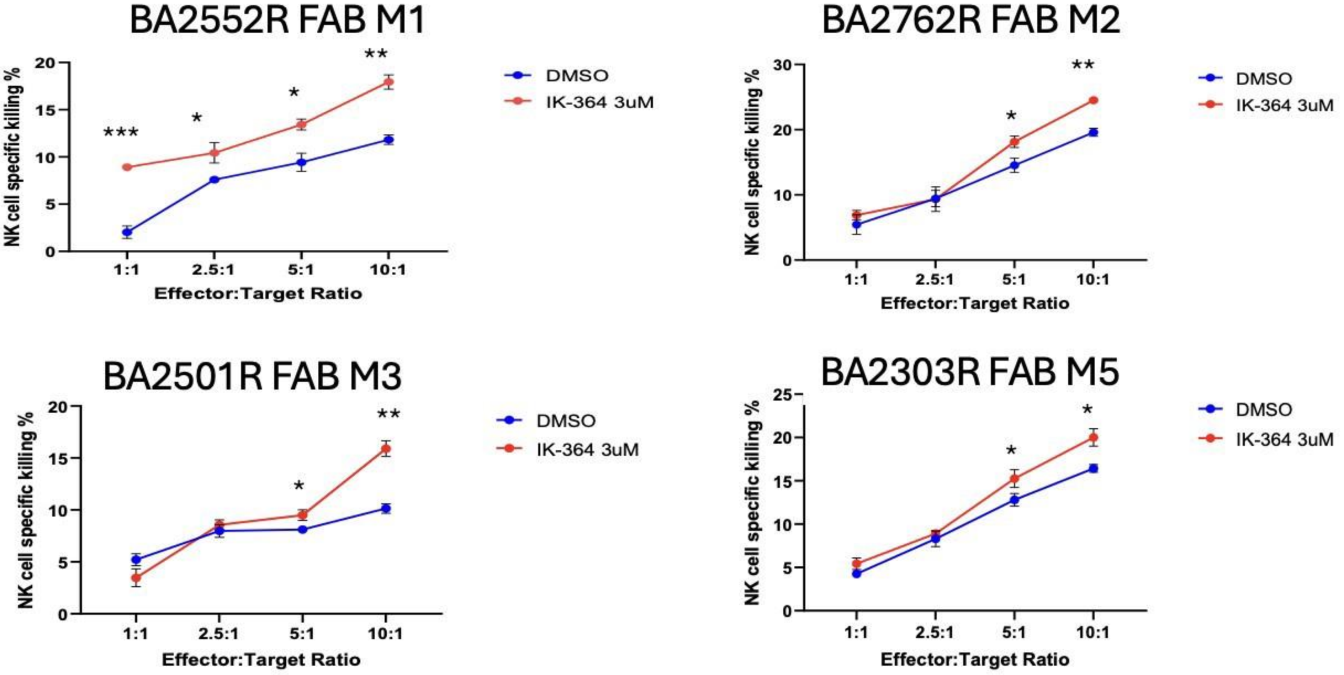

Supplementary Table 5. List of all guides used for CRIPSR genetic knockout experiments.

sgRNA_AHR_A2	caccgTTGCTGCTCTACAGTTATCC
sgRNA_AHR_B2	aaacGGATAACTGTAGAGCAGCAAc
sgRNA_AHR A3	caccgAATTTCAGCGTCAGCTACAC
sgRNA_AHR_B3	aaacGTGTAGCTGACGCTGAAATTc
sgRNA_IFNGR1 G1	caccgATTGTAACATTAGTTGGTGT
sgRNA_IFNGR1 G2	caccgGGTACTCCCAATATACGATA
sgRNA_IFNGR1 G3	aaacACACCAACTAATGTTACAATc
sgRNA_IFNGR1 G4	aaacTATCGTATATTGGGAGTACCc

Supplementary Figure 6. Volcano Plot showing bulk RNA sequencing on 24-hr treated MOLM-14 cells with 3uM IK-364 vs DMSO.

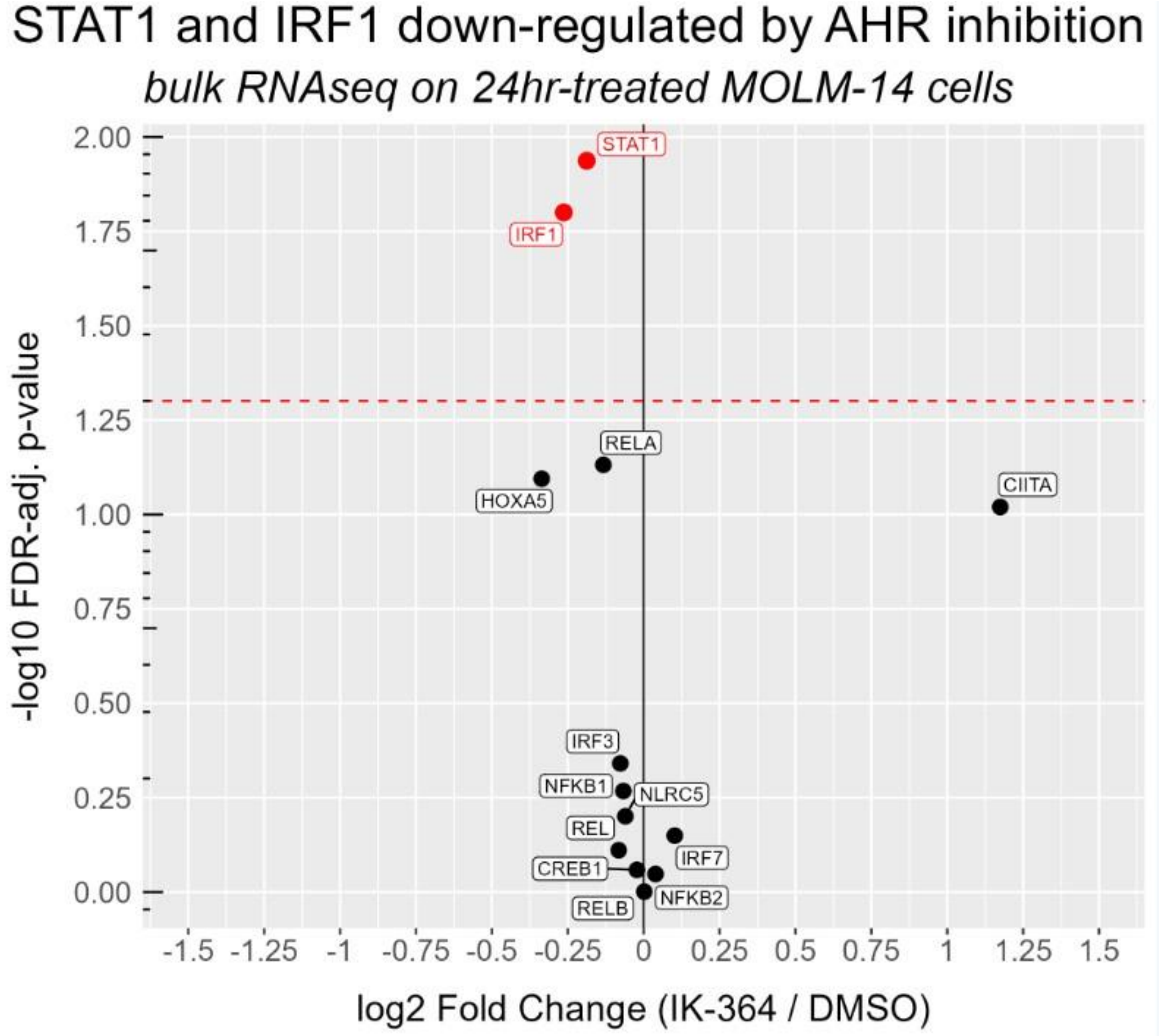

Supplementary Figure 7. A. HLA-E protein expression based on gMFI in double g1g2 AHR knockout MOLM-14 cells compared to non-target (NT) control. B. Western blot showing AHR knockout MOLM14 compared to NT control.

A.

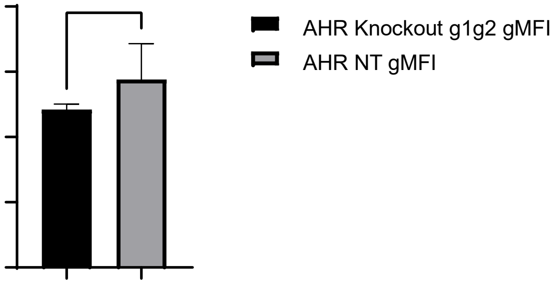

B.

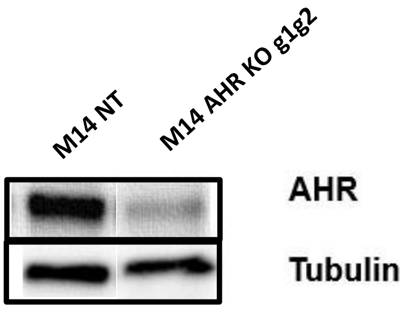

Supplement: Supplementary file 1 [file DataSheet1.pdf]
